# Use of an Individual-based Model to Control Transmission Pathways of *Mycobacterium avium* Subsp. p*aratuberculosis* Infection in Cattle Herds

**DOI:** 10.1038/s41598-017-12078-z

**Published:** 2017-09-19

**Authors:** M. A. Al-Mamun, R. L. Smith, Y. H. Schukken, Y. T. Gröhn

**Affiliations:** 1 Department of Population Medicine and Diagnostic Sciences, Cornell University, College of Veterinary Medicine, Tower Road, Ithaca, New York, 14853 United States of America; 2 Department of Pathobiology, University of Illinois, College of Veterinary Medicine, Urbana, Illinois 61802 United States of America; 3 GD Animal Health, Arnsbergstraat 7, 7411 EZ Wageningen, The Netherlands; 4 Department of Animal Sciences, Wageningen University, Wageningen, 6700 AH The Netherlands

## Abstract

Johne’s disease (JD) is a chronic enteric disease in cattle caused by *Mycobacterium avian* subsp. *paratuberculosis* (MAP). Eradicating JD is a difficult task due to the long incubation period of MAP, inefficient diagnostic tests, and delayed clinical signs. Effective control strategies can help farmers to reduce prevalence, but those most acceptable to farmers combine specific information about lactation performance and testing results, which existing models do not provide. This paper presents an individual-based model of MAP infection dynamics and assesses the relative performance of the applied alternative control strategies. The base dairy herd model included the daily life events of a dairy cow and reflects several current dairy management processes. We then integrated MAP infection dynamics into the model. The model adopted four different test-based control strategies based on risk-based culling decisions and three hygiene scenarios. The model tracked the source of each infection and quantified the efficacy of each control strategy in reducing the risks of different transmission routes. The results suggest that risk-based culling can reduce prevalence compared with no control, but cannot eliminate the infection. Overall, this work provides not only a valuable tool to investigate MAP transmission dynamics but also offers adaptability to model similar infectious diseases.

## Introduction

Johne’s disease (JD), caused by *Mycobacterium avium* subspecies *paratuberculosis* (MAP), is a chronic disease known to affect domestic and wild ruminants. A survey showed that 68% of US dairy herds have at least one MAP infected cow^1^. The economic burden is estimated to be more than $200 million per year due to reduced milk production and pregnancy rates, increased culling rates and reduced slaughter value^2–6^. Control strategies can reduce the endemic prevalence, but the cost of control can also be high^7^. In addition, MAP may potentially have a role in the pathogenesis of Crohn’s disease in humans^8,9^.

The primary route of MAP infection is faecal-oral and calves are more prone to acquire infections during their calfhood^8^. Calves become infected by direct or indirect contact with the pathogen, either horizontally (i.e., contaminated teats, colostrum/milk, pasture, feed, soil, water, and other surfaces) or vertically (in utero)^10^. In adult animals, ingestion of MAP does not necessarily lead to infection, but repeated uptake of high doses of bacilli may result in adult infection^11,12^. In recent studies, adult-to-adult, calf-to-calf, and heifer-to-heifer infections have been shown to exist^13,14^. Some animals may exhibit progressive clinical disease, while others remain subclinical long-term, but the mechanism that distinguishes between these two pathways is still unknown^15^.

From the farmer’s point of view, JD is a difficult disease to diagnose as there are often no clinical signs in adult animals even several years after initial infection. Inefficient testing methods with low test sensitivity and infrequent testing also make it more difficult to identify the truly infected animal^16^. Currently, there is no treatment available for JD, but multiple control strategies and certification programs have been adopted in several countries with limited success^5,17,18^. One way to reduce the prevalence is to cull shedders or clinical animals, as this may decrease the number of transmission events^19^. However, culling low shedders is often not economically desirable due to low clinical disease risk and unaffected fertility and milk production^3^. Culling high shedding animals may reduce infection pressure, but it is difficult and expensive for a farmer to identify which animals to cull. Given all these limitations in implementing control programs, it is important to provide realistic decision support for a farmer wishing to control MAP through culling.

Mathematical simulation models have been developed to recreate MAP infection dynamics and to understand the impact of different actions on the endemic infection level of MAP in the herd^15,17,20–23^. However, most previous models are compartmental and have implemented broad control strategies such as generalised test-and-cull of adult animals and improved calf rearing management, decreasing MAP transmission routes in young susceptible calves^19,24^. These programs aimed at reducing risk factors for MAP transmission^18,25^. Although previous compartmental models have provided a substantial improvement in our understanding of MAP infection dynamics at the herd level, detailed information about individual animals can be more useful to the farmer. Recently, an individual-based modelling approach has been adopted to build a dairy herd model^26–28^. However, none of these published models considered alternative risk-based (targeting animals based on risk groups) culling strategies to impact different MAP transmission routes. This motivated us to build an individual-based model (IBM) to quantify the relative assessment of test-based control strategies based on risk-based culling while considering individual animal information on a daily basis.

The main objective of this paper was to perform simulations studies using an IBM of a dairy herd incorporating MAP infection dynamics to determine the relative efficacy of test-based control strategies based and risk-based culling on different transmission routes. Here, we present an IBM for a closed dairy herd (no new animals are bought from outside) where each individual animal is assigned its own demographic characteristics, reproduction status, and infection status. The model integrates three management hygiene strategies and considers four MAP control strategies aimed at affecting the MAP transmission routes.

## Methods

The model formulation and simulation is described by the standard IBM protocol ODD (overview, design concepts, and details) suggested by Grimm^29^. The ODD protocol describes an individual model in terms of seven sections: (i) purpose, (ii) state variables and scales, (iii) process overview and scheduling, (iv) design concepts, (v) initialization, (vi) input, and (vii) sub-models. The basis of this individual-based dairy herd model has been described previously^28^.

### Purpose, State Variables, and Scales

**Figure 1:**
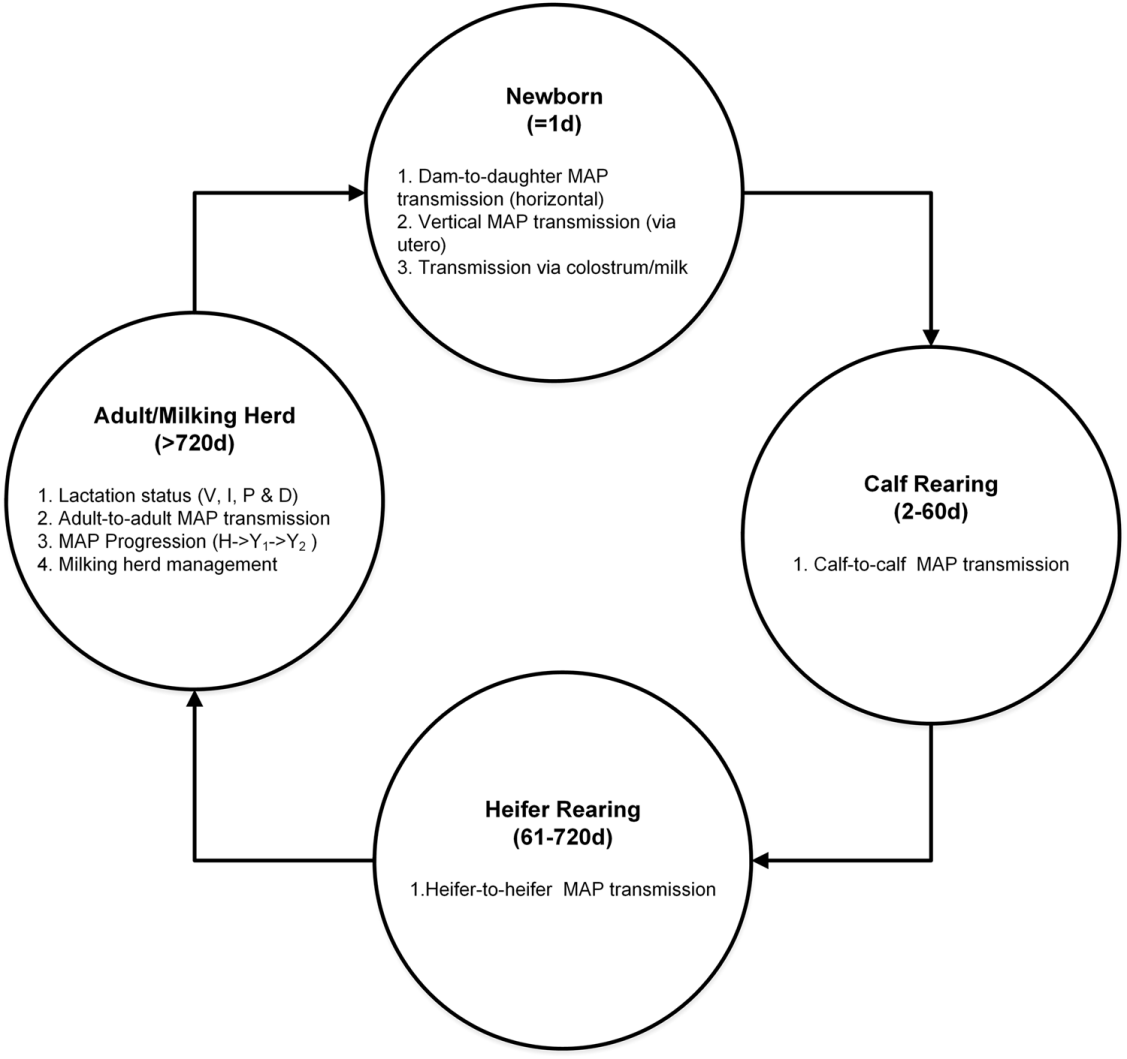
The overview of a typical dairy herd in terms of management groups. Adult animals resided in adult/milking herd group (left circle), after calving, newborns remained with the dam in maternity pen for one day (top circle), then calves older than one day transferred to the calf rearing group (right circle) and after 60 days they transferred to the heifer rearing group (bottom circle). Six transmission routes were considered in the model. There were four lactation states (V-voluntary waiting period, I-insemination, P-pregnancy, and D-dry-off-period) and three MAP progression states (H-latent, Y_1_-low shedding, Y_2_-high shedding animals). Abbreviation: d: days.

The purpose of the model was to develop an IBM that would inform farmers which control strategy is appropriate to stop or break different MAP transmission routes in a dairy herd using the information of individual animals. Several simulation experiments were designed to demonstrate the value of individual animal-level information in decision making. The model defined an individual as a bovine (newborn animal, calf, heifer or an adult cow) to be called a cow throughout this manuscript. Each cow was assigned inherent demographic and infection properties based on its age. A cow was in four age-based management groups (shown in Fig. 1): newborn, calf rearing, heifer rearing, and the adult/milking herd. In this model, a cow had an integer state variable *age* and several binary states: *lactation status*, *infection status*, and *enzyme-linked immunosorbent assay* (*ELISA) testing results*. The milking herd was tested periodically for MAP infection and based on current and previous testing results each adult cow was identified as belonging to one of three risk categories: red, yellow, and green (see subsection ‘*Testing and Risk-based Control Strategies*’). Red or contagious animals were prioritised for culling based on the control strategies. A cow was culled due to death or management-related reasons through an age-dependent stochastic process^28^. All lactation status variables depended on lactation process sub-model completion. All state variables (shown in Supplementary Table S1) could change over time. The model had two-time scales: individual animal age and simulation time step. In both cases, each time step represented 1 day.

### Process Overview and Scheduling

During each time step, each cow went through three processes: lactation and birth, infection transmission, and testing and control. These three processes involved several functions which are described in the ‘*Sub-models (Functions)*’ section. To complete a model time step, first, the probability of giving birth was checked. If a new calf was born, it was transferred to the calf rearing group after one day and the cow’s lactation state was updated. Then, the infection transmission probability was checked and the cow’s MAP state was updated as needed. Finally, a cow was tested for MAP depending on the herd time and testing frequency and each tested cow was kept or culled based on the outcome of the test and the particular control scenario being modelled. The lists of the performed processes are displayed in the Supplementary Information (see Supplementary Fig. S1).

**Figure 2:**
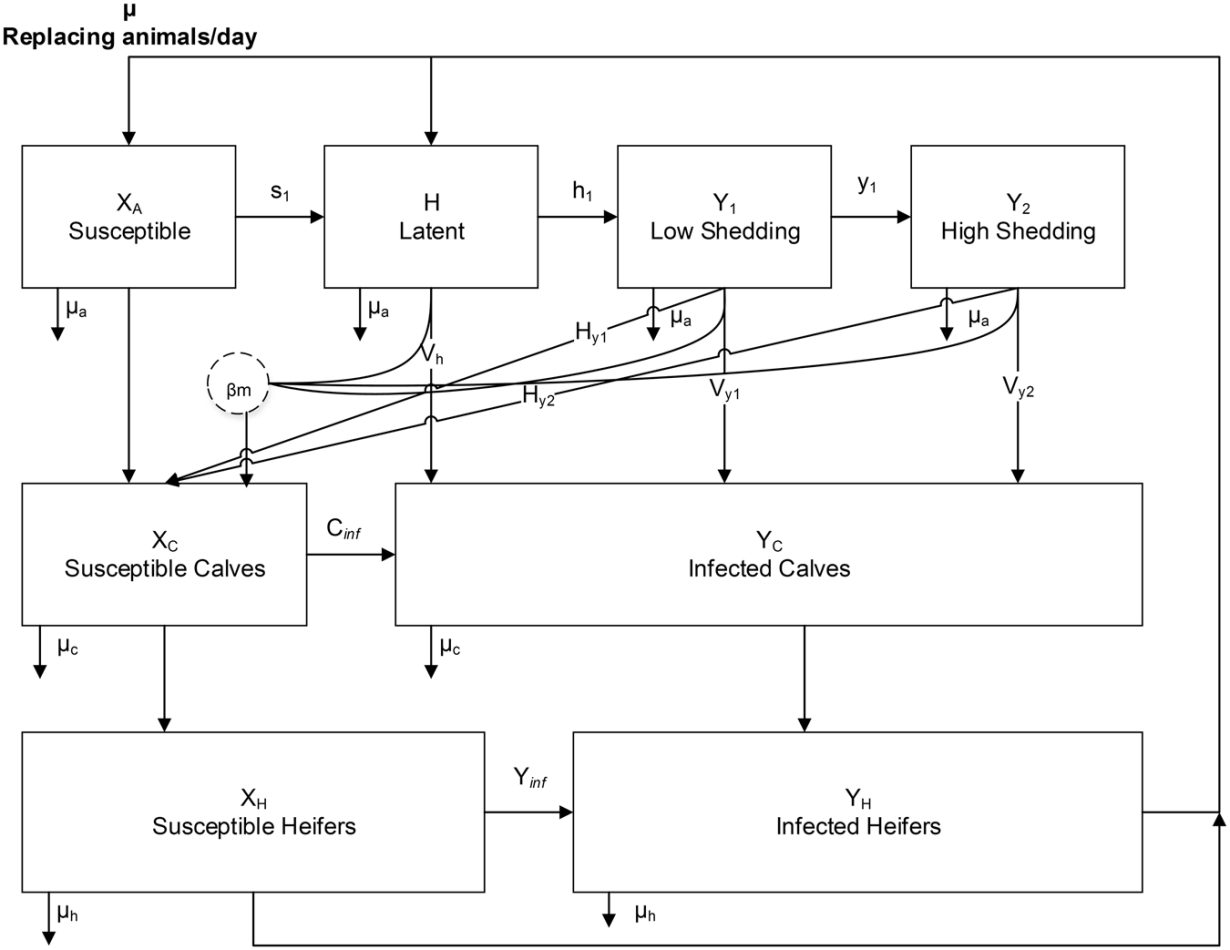
A flow diagram of infection categories for the adult animals in the herd. Each category (boxes) classifies animals according to their initial setup. The probabilities of exit at each time point from susceptible to latent, latent to low shedding and low shedding to high shedding animals are s_1_, h_1_, and y_1_, respectively. Vertical transmission probabilities from latent, low shedders and high shedders animals are V_h_,V_y1_, and V_y2_, respectively. Horizontal transmission probabilities to calves from low shedders and high shedders animals are H_y1_ and H_y2_, respectively. The probability a calf is infected by colostrum/milk from infected animals is *β*_*m*_. Calf-to-calf and heifer-to-heifer transmission probabilities are C_*inf*_ and *Y*_*inf*_, respectively. Stochastic death/sale probabilities for adult, calves, and heifers are µ_a_, µ_c,_ and µ_h,_ respectively.

The infection compartments in the milking herd were divided into four categories: Susceptible (X_A_), latent (H), low shedding (Y_1_), and high shedding (Y_2_). In calf rearing housing, there were two infection categories: susceptible (X_C_) and infected (Y_C_). In heifer rearing housing, there were also two infection categories: susceptible (X_H_) and infected (Y_H_). We included six different transmission routes: adult-to-adult, dam-to-daughter (vertical), dam-to-daughter (horizontal transmission from environmental contamination as a newborn), colostrum/milk (adult-to-calf), calf-to-calf, and heifer-to-heifer. The detailed infection structure is shown in Fig. 2.

### Design Concepts

#### Emergence

**‘**Emergence’ describes what key results or outputs of the model are modelled as emerging from the adaptive traits, or behaviours, of individuals. The model shows the impact of test and risk-based culling strategies on endemic herds. Population dynamics emerge from the individual animal’s behaviour. Three layers of dynamics have been integrated into the model: individual animal lactation, MAP transmission and herd management with testing, and test-based control strategies. Individual behaviour was set according to empirical rules and maintained throughout the simulation period, whereas infection dynamics relied on different infection routes in different stages of the cow’s life.

#### Sensing

Individual cows were assumed to “know” all their inherent characteristics and to update those characteristics after every simulation time step. Individuals behaved according to their lactation and infection states.

#### Interaction

Several interactions among individuals were modelled. First, each susceptible adult, heifer, or calf could be infected horizontally by low and high shedding animals with a probability based on age group. Second, a newborn calf could be infected in three ways: vertically from an infected dam via in-utero infection, horizontally from the infected adults in the calving pen on the day it was born, and pseudo-vertically by colostrum/milk from infected dams. Third, progression between the three age groups ensured that MAP transmission to calves or heifers resulted eventually in infected adults. For horizontal infection routes, individuals were assumed to interact indirectly through the faecal-oral transmission.

#### Collectives

The individuals were grouped into three collective groups during the simulation period: management groups based on age, infection status groups, and risk groups based on testing strategy. The management groups were determined by the age of the individual animals (as described in Fig. 1). The age groups were divided into adults (>720 days), newborn (1 day), calves (2–60 days), and heifers (61–720 days). The management groups were used to create the initial populations. Infection states influence infection dynamics over the entire population. The testing groups (green, yellow, and red) were created to implement risk-based culling decisions.

#### Stochasticity

Several processes were designed as stochastic processes, such as individual mortality, assignment of calf gender, disease progression, infection routes (adult-to-adult, dam-to-daughter (vertical), dam-to-daughter (horizontal), colostrum/milk (adult-to-calf), calf-to-calf, and heifer-to-heifer), insemination success, and sensitivity, and specificity of diagnostic tests. The initial age distribution was randomly assigned based on a given parity distribution.

#### Observation

At each time step, several variables were recorded: prevalence, the number of milk ELISA tests completed and their results, risk-based animal distribution, the number of dead animals, the number of replacements, the number of animals passing through each transmission route, and the number of available calves and heifers. All individuals were tracked at each time step.

### Initialization

The model was initialised with 200 adult animals distributed into parity 1 (45%), parity 2 (25%), and parity 3 and above (30%). Initially, each animal was randomly assigned an age according to her parity, then lactation stage was determined from the given age. Before introducing any infected animals into the herd, the model was run until empirically stable conditions were achieved. After obtaining a stable disease-free herd, we then introduced infected animals from a predefined distribution. All simulations were run for 17 years after the introduction of infected animals, with the first two years discarded as burn-in time; 3 five year windows were identified to assess the comparative effectiveness of the alternative control programs^18^. Initial parameter values were informed by data obtained from observational studies such as the longitudinal Regional Dairy Quality Management Alliance (RDQMA) study as described previously^16^ (see Supplementary section A for details).

### Input

The herd size of 200 adult animals was chosen according to the average of a medium-sized US herd^30^. We simulated three MAP infection prevalence levels to represent three common farm hygiene levels: 5–15% (moderate), 15–25% (low), and 30–40% (poor or ‘no hygiene policy’). After producing a stable endemic herd for each scenario, we simulated different testing and control scenarios. Further details of all parameters used during model construction are provided in the Supplementary Information (see Supplementary Table S2).

### Sub-models (Functions)

#### Lactation status

In each day of the simulation, the model checked each individual’s lactation status to determine the sequential group of life events. The lactation status function had four different subroutines: voluntary waiting period (VWP), inseminations, pregnancy, and dry-off period. The VWP started immediately after calving and lasted 60 days. In this period, adults are not inseminated even if they display estrus, to allow for optimum uterine involution, and recovery from negative energy balance. Once the VWP had passed, an animal was inseminated repeatedly until a successful conception. If the animal conceived after insemination, it passed to pregnancy status at 21 days, otherwise, inseminations continued until 225 days in milk (DIM). If the animal did not become pregnant by 225 DIM, it was culled as an open cow. The dry-off period began 60 days prior to calving. During this time period, milk production was halted. Once the animal was ready to calve, the flow was transferred to another routine called ‘Birth’. More details about the lactation cycle can be found in the study by Al-Mamun *et al*.^28^.

#### Birth, survival and additional culling

The birth routine checked the probability of producing a female calf for each pregnant dam. Only female offspring were kept in the herd; male off-spring were removed immediately after birth. This function also checked the probability of survival for each individual animal. The ‘additional culling’ routine was called when the model marked a cow as green, yellow or red. This routine kept the green and yellow animals and culled the red animals according to the adopted control schemes.

#### Milking herd group

In the milking herd group, adult animals could be infected by low and high shedding adults. The transmission probability of faecal-oral transmission for adult animals can be given by:

1 $$In{f}_{adult{\mbox{--}}adult}=\frac{1}{{G}_{i}}(\frac{{\beta }_{A}({\beta }_{{Y}_{1}}{Y}_{1}+{\beta }_{{Y}_{2}}{Y}_{2})}{N}),i=1({\rm{m}}{\rm{o}}{\rm{d}}{\rm{e}}{\rm{r}}{\rm{a}}{\rm{t}}{\rm{e}}\,{\rm{h}}{\rm{y}}{\rm{g}}{\rm{i}}{\rm{e}}{\rm{n}}{\rm{e}}),2({\rm{l}}{\rm{o}}{\rm{w}}\,{\rm{h}}{\rm{y}}{\rm{g}}{\rm{i}}{\rm{e}}{\rm{n}}{\rm{e}}),3({\rm{p}}{\rm{o}}{\rm{o}}{\rm{r}}\,{\rm{h}}{\rm{y}}{\rm{g}}{\rm{i}}{\rm{e}}{\rm{n}}{\rm{e}})$$

Susceptible adult animals in the milking herd compartment were susceptible to MAP infection by contact with low shedding (Y_1_) and high shedding (Y_2_) animals with transmission rates of ${\beta }_{{Y}_{1}}$ and ${\beta }_{{Y}_{2}}$, respectively. ${\beta }_{A}$ is the adult-to-adult transmission coefficient and N is the total number of animals in the milking herd, N = *X*_*A*_ + *H* + *Y*_*1*_ + *Y*_*2*_. The value of the hygiene parameter (*G*_*i*_) was chosen such that for poor hygiene, the risk of transmission was higher than moderate and low hygiene. The daily horizontal infection probability to calves can be determined by

2 $$In{f}_{adult{\mbox{--}}calf}=\frac{1}{0.1\times {G}_{i}}(\frac{{\beta }_{a}({\beta }_{{Y}_{1}}{Y}_{1}+{\beta }_{{Y}_{2}}{Y}_{2})}{{N}_{c}})$$

${\beta }_{a}$ is the horizontal transmission coefficient for an adult to newborn calves and *N*_*c*_ is the total number of calves at every day, *N*_*c*_ = *X*_*c*_ + *Y*_*c*_. G_i_ is the hygiene parameter described in equation 1.

#### Calf rearing group

A calf stayed in calf rearing housing for the first 60 days after birth. During this period, she could get infected indirectly by colostrum/milk from infected dams and directly by other infected calves. The probability of direct transmission was calculated as

3 $$In{f}_{calf{\mbox{--}}calf}=\frac{1}{{G}_{i}}(\frac{{\beta }_{a}{Y}_{c}}{{N}_{{X}_{c}}})$$

${\beta }_{a}$ is the horizontal calf-to-calf transmission coefficient, ${N}_{{X}_{c}}$ is the total number of calves at each day, *X*_*c*_ is susceptible calves, *Y*_*c*_ is infected calves. ${N}_{{X}_{c}}={X}_{c}+{Y}_{c}$ and *G*_*i*_ is the hygiene parameter described in equation 1. During the first day after birth, a calf may also be infected horizontally by infected adults present in the maternity pen or vertically by an infected dam.

#### Heifer rearing group

Animals aged 2–24 months were placed in the heifer rearing group. At 15 months of age, each heifer was inseminated for the first time; the estimated entry point into the milking herd group was assumed to be one week before calving. Susceptible calves became susceptible heifers and infected calves became infected heifers. Infected heifers could infect susceptible heifers by the heifer-to-heifer transmission path:

4 $$In{f}_{heifer{\mbox{--}}heifer}=\frac{1}{{G}_{i}}(\frac{{\beta }_{h}{Y}_{H}}{{N}_{{X}_{H}}})$$

${\beta }_{h}$ is the horizontal heifer-to-heifer transmission coefficient, and the total number of heifers is ${N}_{{X}_{H}}={X}_{H}+{Y}_{H}$. After one year, the infected heifers became latent heifers and eventually entered the milking herd as latent adults. G_i_ is the hygiene parameter described in equation 1.

#### Adult progression

The ‘Adult progression’ routine describes the progression through the three different infection groups: latent, low shedders, and high shedders. Latent adults could progress to low shedding status and low shedding adults could progress to high shedding status based on their respective progression probability. High shedding adults could not progress further and their infection status remained the same through the rest of their life. The pseudocode for all these sub-models is presented in supplementary section A. A flow chart for all sub-models can be found in the Supplementary Information (see Supplementary Fig. S2).

### Testing and Risk-based Control Strategies

Several experimental herds were developed based on hygiene practices: moderate, low, and poor hygiene. Each herd was simulated for three testing frequencies: annual, bi-annual, and quarterly. In the current study, we simulated only the milk antibody ELISA for testing of adult animals. The sensitivity and specificity of the milk ELISA, stratified by parity, and DIM, is described in the Supplementary Information^31^ (see Supplementary Table S3). This model categorised adults into three different risk groups based on testing results. All cows that tested negative throughout testing were marked as low risk or green cows. The cows that tested positive were divided into two groups: yellow and red. Red animals had at least 2 positive tests out of the last 4 tests and yellow cows had one positive test. This color coding scheme was modified from Kudahl *et al*.^31^. The model considered the no control and four risk-based culling strategies and compared the control strategies using ONEWAY ANOVA tests followed by a post-test Bonferroni to determine the significant control strategy. The control strategies such as

1. No control: No MAP-related culling was adopted. The dairy herd started from the initially infected animals and hygiene level.
2. Control I (aggressive culling): Red cows were culled immediately after identification, regardless of their lactation status and DIM.
3. Control II (culling open red cows after 305 DIM): If red cows were in the VWP or waiting to be inseminated, they were not to be inseminated and would remain in the herd up to 305 DIM before culling. During this period, the red flagged animals resided in the same barn with other animals. Pregnant red cows were managed as in the no control strategy.
4. Control III (culling dam and offspring): Pregnant red cows were kept until the end of the pregnancy and at that point, dam and offspring (male or female) were culled.
5. Control IV (culling dam but not the offspring): Same as control III except the female newborn calf was not culled; male calf and the dam were culled.

### Global Sensitivity Analysis

We selected 15 parameters $({\mu }_{a},{\mu }_{c},{\mu }_{h},{\beta }_{A},{\beta }_{a},{\beta }_{c},{\beta }_{m},{\beta }_{h},{\beta }_{{Y}_{1}},{\beta }_{{Y}_{2}},$ ${V}_{h},{V}_{{Y}_{1}},{V}_{{Y}_{2}},{y}_{1}\,{\rm{and}}\,{h}_{1})$ to carry out a global sensitivity analysis for prevalence over time, as these parameters contain some degree of uncertainty. We explored the parameter space by using the Latin Hypercube Sampling (LHS) method and evaluated partial rank correlation coefficients (PRCCs) with prevalence at year 5^22,28,32^. The parameters displayed in the Supplementary Tables S2 and S4 were varied by 50% of their base value. First, we drew 100 samples for each parameter within the suggested range using random LHS (RLHS); RLHS spreads the sample points more evenly across all possible values and selects a random point within each interval. Then, we ran 100 replicates of IBM for each set of parameters. For all the other scenarios, we also ran 100 replicates of the IBM.

## Results

### Background Simulation and Tracking

First, the IBM was simulated in a disease free condition to evaluate whether our baseline model performed like a realistic dairy herd. Table 1 presents the distribution of key measurements as simulated by the model of the uninfected herd. These measures resemble those parameters observed in Dairy Management-UW Extension and 5 commercial dairy herds in New York State^33–35^. The parameter values used during this simulation can be found in the Supplementary Information (see Supplementary Table S4).

**Table 1:** Statistics of underlying processes of an uninfected stable herd.

Herd factors	Simulated values Mean (95% CI)	Observed values Mean (95% CI)
Number of animals	217 (213–221)	NA^a^
Number of available replacements	137 (133–141)	NA^a^
Calving interval (days)	410 (400–420)	409.6 (408.2–411.1)^b^
Success of inseminations (attempts)	3.1 (3.07–3.14).	2.93 (2.90–2.95)^b^
Annual pregnancy percentage (%)	71.1 (70.8–71.5)	73.5 (72.0–74.8)^b^
Annual involuntary culling (%)	8.0 (7.9–8.1)	15.3 (14.8–15.7)^b^
Annual voluntary culling (%)	31.7 (31.6–31.9)	51.0 (50.4, 51.6) ^b^

^a^The observed values can be obtained from www.dairymgt.info/tools/heifer_replacement/index.php heifer replacement tool developed by University of Wisconsin Dairy management group. ^b^The observed values were calculated from data collected from 5 New York herds from 2003–2004 until 2011.

Second, the model was simulated with MAP dynamics prior to implementing any intervention strategies. Transmission parameters for the three hygiene scenarios were chosen using a parameter search space and calibration method such that the infection would persist in the herd at different levels (see Supplementary section B). The true prevalence of the three scenarios after 15 years of simulations was 10.2 (95% CI: 9.3–11.0), 22.5 (95% CI: 21.0–23.9) and 34.7 (95% CI: 32.9–6.4) for moderate, low and poor hygiene, respectively (see Supplementary Fig. S3). The parameter space search and model are described in supplementary section B. Supplementary Table S5 provides the ranges of selected parameters that were calibrated during the parameter search.

Third, each individual animal was tracked from birth to death/culling to record relevant information for infection dynamics. Supplementary Fig. S4 describes a tracking example of four different animals, each with a different infection status (X_A_, H, Y_1_, and Y_2_) till the end of their life.

### Evaluation of Control Efficacy

**Figure 3:**
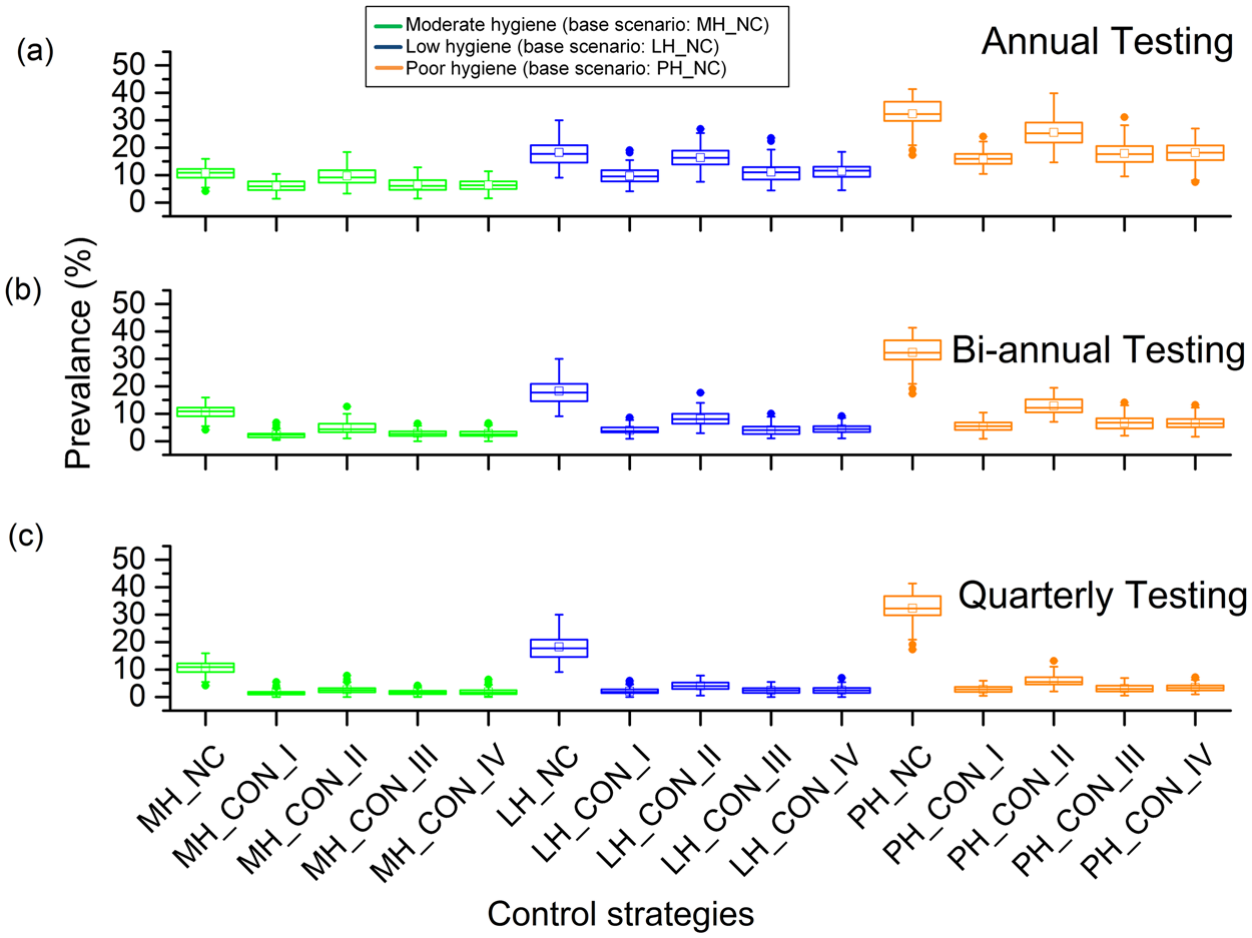
Evaluation of control strategies in three hygiene conditions at fifth year for (**a**) annual testing, (**b**) bi-annual testing, and (**c**) quarterly testing. In all box plots, the bottom and top end of the bars are minimum and maximum values respectively, the top of the box is the 75th percentile, the bottom of the box is the 25th percentile, and the horizontal line within the box is the median; outliers are presented as a solid circle. Abbreviations:, MH_NC: moderate hygiene-no control, MH_CON_I: moderate hygiene-control I, MH_CON_II: moderate hygiene-control II, MH_CON_III: moderate hygiene-control III, MH_CON_IV: moderate hygiene-control IV, LH_NC: low hygiene-no control, LH_CON_I: low hygiene-control I, LH_CON_II: low hygiene-control II, LH_CON_III: low hygiene-control III, LH_CON_IV: low hygiene-control IV, PH_NC: poor hygiene-no control, PH_CON_I: poor hygiene-control I, PH_CON_II: poor hygiene-control II, PH_CON_III: poor hygiene-control III, PH_CON_IV: poor hygiene-control IV.

We ran the model for three different hygiene scenarios (moderate, low, and poor) and 4 different risk-based control strategies (control I, II, III & IV) at 3 different testing frequencies (annually, bi-annually, and quarterly) to see whether the selected test-based control strategies could reduce prevalence or eradicate MAP. All control strategies were compared to the scenario with no control, which had a median prevalence after 5 years of 10.9%, 17.7%, and 32.2% for moderate, low, and poor hygiene, respectively. Figure 3a presents the annual test-and-cull results based on 4 different control strategies. The median result from aggressive culling was best among all controls, reducing prevalence by 44% in the fifth year, depending on hygiene level. Culling dam and offspring (control III) and culling dam not the offspring (control IV) were not significantly different from culling open red cows after 305 DIM (control II), reducing prevalence after 5 years by 39% and 37%, respectively (see Supplementary Table S6). For annual and bi-annual testing, culling open red cows after 305 DIM (control II) performed poorly compared to other control strategies, but for quarterly testing, it did not differ from other controls.

For bi-annual testing and risk based culling (see Fig. 3b), culling dam and offspring (control III) and culling dam not the offspring (control IV) were again not significantly worse than aggressive culling (control I) in the long term (10 and 15 years) (see Supplementary Figs S5 and S6), but aggressive culling (control I) was significantly better in the first 5 years in all but low hygiene herds (see Supplementary Tables S7 and S8). For quarterly test-based control strategies (see Fig. 3c), aggressive culling (control I) and culling dam and offspring (controls III) were not significantly different and produced the lowest prevalence over 5 years and 15 years (see Supplementary Tables S6 and S8). Also, culling open red cows after 305 DIM (control II) performed like other controls in a quarterly test setting. However, MAP was not eradicated in any scenario due to persistent infections in calf rearing and heifer management.

### Evaluation of Dam-to-Daughter Transmission (Horizontal and Colostrum/Milk)

**Figure 4:**
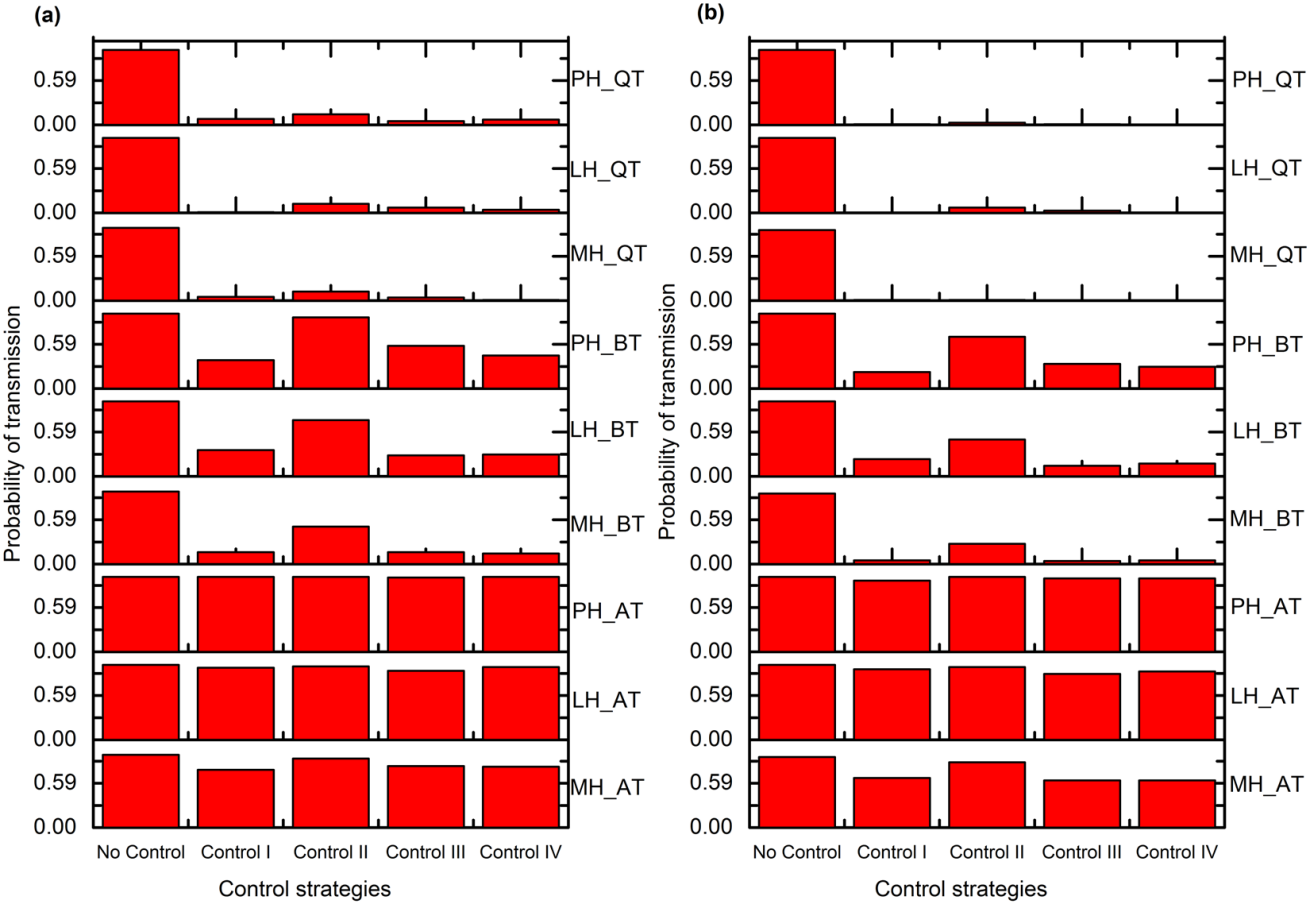
The probability of transmission by dam-to-daughter (**a**) and colostrum/milk (**b**) transmission routes after implementing the control strategies on 9 different herd scenarios. Abbreviations: MH_AT: moderate hygiene-annual testing, LH_AT: low hygiene-annual testing, PH_AT: poor hygiene-annual testing, MH_BT: moderate hygiene-bi-annual testing, LH_BT: low hygiene-bi-annual testing, PH_BT: poor hygiene-bi-annual testing, MH_QT: moderate hygiene-quarterly testing, LH_QT: low hygiene-quarterly testing, and PH_QT: poor hygiene-quarterly testing.

In this model, there are two main dam-to-daughter horizontal transmission paths: direct infection in the maternity pen immediately after birth, and infection by colostrum/milk from shedding animals. Figure 4a shows the probability of dam-to-daughter horizontal transmission for each control scenario. When quarterly testing was applied, aggressive culling (control I) decreased the probability of dam-to-daughter transmission by 93% by the 5^th^ year of the intervention compared to no control, while culling dam and offspring (control III) decreased the probability of dam-to-daughter transmission by 97% over the same time period. Culling open red cows after 305 DIM (control II) performed poorly for annual and bi-annual testing but was considerably effective with quarterly testing. Culling dam not the offspring (control IV) showed rare infection events of vertical transmission even with quarterly testing, likely due to calves from infected dams raised as replacements. Figure 4b shows the probability of transmission through the colostrum/milk infection route; with quarterly testing, it is unlikely to observe any transmission over this route after 5 years under aggressive culling (control I) or culling open red cows after 305 DIM (control II) for any hygiene scenario. No control strategy performed well with annual testing, and poor hygiene decreased the effectiveness of test-and-cull programs even with quarterly testing. Aggressive culling (control I) was the best option for reducing the number of infections by both transmission routes. Culling open red cows after 305 DIM (control II) did not perform well with annual testing but did perform well with quarterly testing.

### Evaluation of Other Transmission Routes

**Figure 5:**
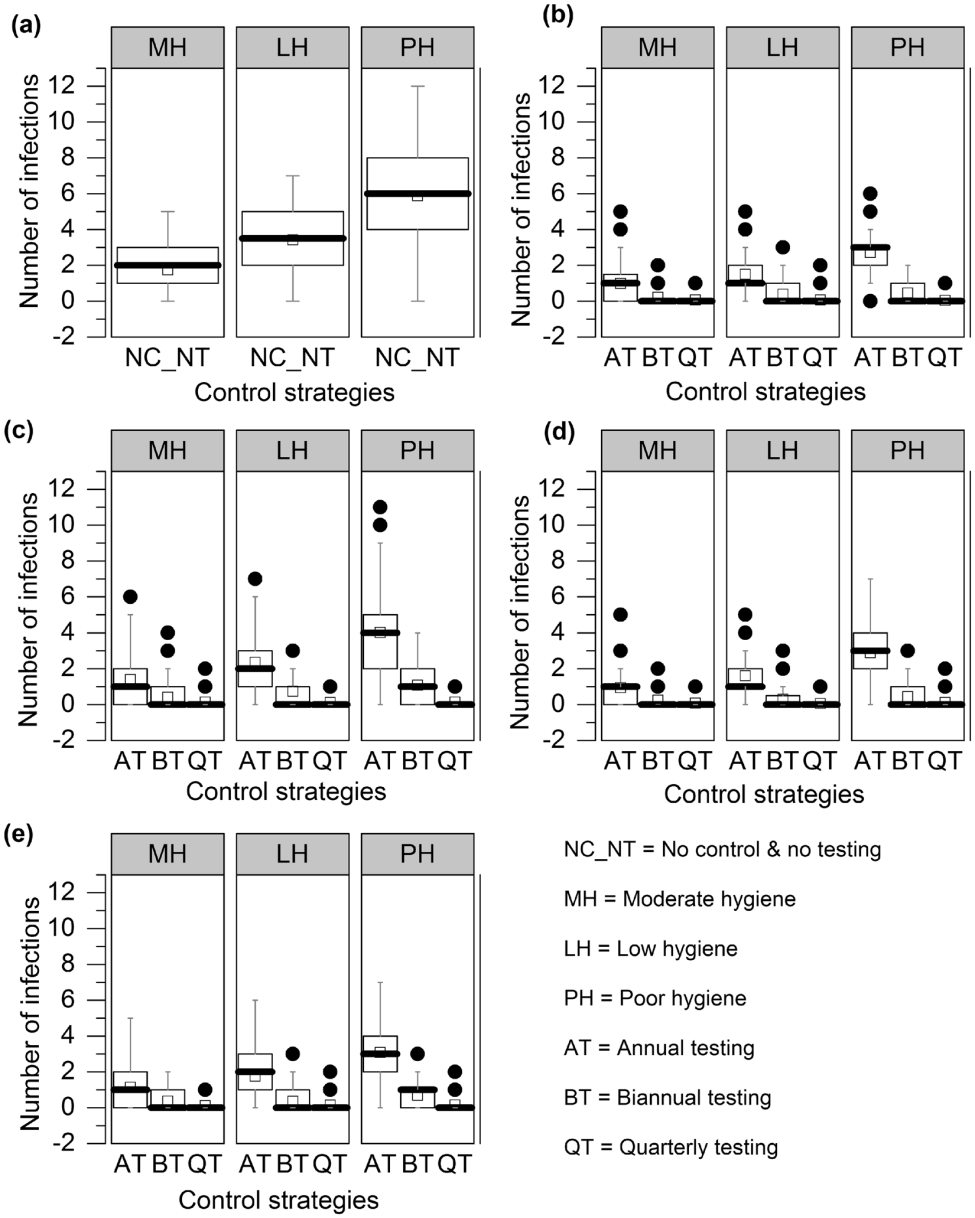
The number of vertical transmission events in 100 simulations at 5th year after the intervention, using (**a**) no control, (**b**) control I (aggressive culling), (**c**) control II (culling open red cows after 305 DIM), (**d**) control III (culling dam and offspring) and (**e**) control IV (culling dam not the offspring) for 9 different hygiene and test frequency scenarios. In the box plot, the bottom and top end of the bars are 5th and 95th percentiles, respectively, the top of the box is the 75th percentile, the bottom of the box is the 25th percentile, the horizontal line within the box is the median, the open square is the mean, and outliers are presented as solid circles.

Figure 5 shows the number of transmission events at the fifth year through the vertical infection route. With no control (see Fig. 5a), vertical transmission shows some infection events, but when serial/quarterly testing was implemented, the average number of vertically infected calves was reduced. The probabilities of infection through vertical transmission for 9 different control scenarios and three testing frequencies are shown in Supplementary Information (see Supplementary Fig. S7).

**Figure 6:**
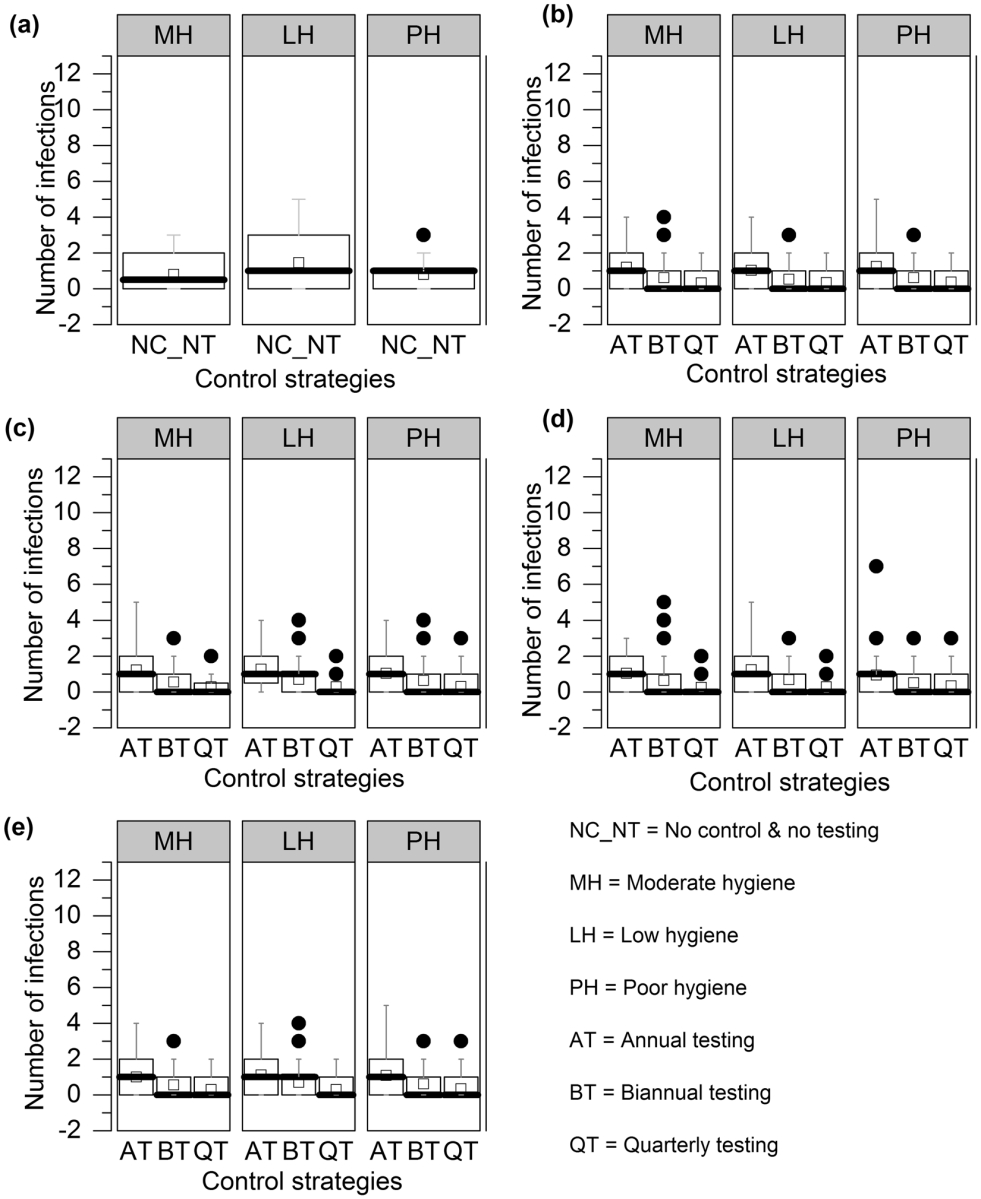
Number of infections events occurring via heifer-to-heifer transmission route in 100 simulations at 5^th^ year while considering, (**a**) no control(**b**) control I (aggressive culling), (**c**) control II (culling open red cows after 305 DIM), (**d**) control III (culling dam and offspring) and (**e**) control IV (culling dam not the offspring)for 9 different scenarios. In the box plot, the bottom and top end of the bars are the 5th and 95th percentiles, respectively, the top of the box is the 75th percentile, the bottom of the box is the 25th percentile, the horizontal line within the box is the median and the open square is the mean; outliers are presented as solid circles.

Figure 6 shows the number of transmission events via the heifer-to-heifer transmission route under different control strategies compared with no control. Few substantial changes were observed for all control programs. This contributed to the disease persistence, as infection could persist in the heifer group despite control in the calf and adult groups. The probabilities of transmission for other control scenarios are described in the Supplementary Information (see Supplementary Fig. S7). The model also recorded the number of transmission events occurring through calf-to-calf and adult-to-adult transmission routes (see Supplementary Fig. S7). Five years after the intervention, all control strategies reduced the calf-to-calf transmission compared to no control, especially with more frequent testing. For adult-adult transmission, however, the probability of infection did not change substantially if only annual testing was conducted.

### Global Sensitivity Analysis

**Figure 7:**
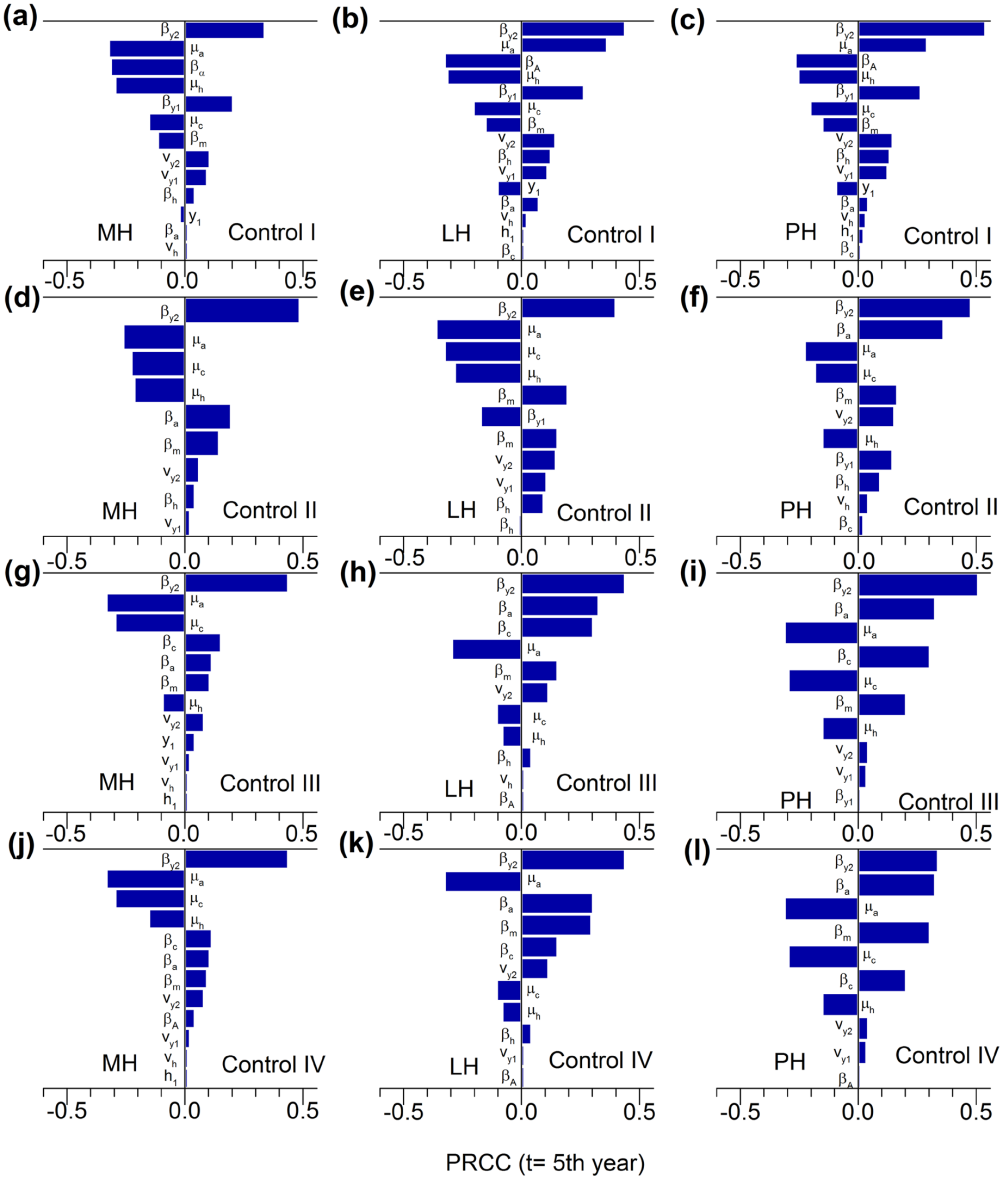
Global sensitivity analysis of true adult prevalence (*H* + *Y*_1_ + *Y*_2_)/*N* for the model parameters while considering four control strategies: control I (aggressive culling), (**c**) control II (culling open red cows after 305 DIM), (**d**) control III (culling dam and offspring) in moderate, low, and poor hygiene herd conditions: (**a**) control I for MH, (**b**) control I for LH, (**c**) control I for PH, (**d**) control II for MH, (**e**) control II for LH, (**f**) control II for PH, (**g)** control III for LH, (**h**) control III for LH, (**i**) control III for PH, (**j**) control IV for MH, (**k**) control IV for LH, and (**l**) control IV for PH Parameters were only shown if their values of PRCC differed significantly from zero (*p value* < 0.01). Abbreviations: MH: moderate hygiene, LH: low hygiene, PH: poor hygiene, PRCC: partial rank correlation coefficients.

The PRCCs for true adult prevalence 5 years after the intervention and selected model parameters are shown in Fig. 7. The most influential parameter on true prevalence for all control scenarios was transmission rate of high shedders (*Y*_2_), ${\beta }_{{Y}_{2}}$. The death rate for adults (*µ*_*a*_) was the second most influential parameter for low hygiene regardless of the control program and was highly influential in all other control scenarios. Other parameters like transmission rate of low shedders (*Y*_1_), ${\beta }_{{Y}_{1}}$, the probability of transmission via colostrum/milk, *β*_*m*_, and the adult-to-adult transmission coefficient, *β*_*A*_, were moderately influential for all control programs. For culling dam and offspring (control III) and culling dam not the offspring (control IV), in which calves born from red dams remained in the herd, the calf-to-calf transmission coefficient, *β*_*c*_, became influential.

## Discussion

In this study, we have presented an IBM to assess the relative efficacy of test-based control strategies based on high-risk animals and different testing frequencies on MAP transmission routes. We have found that all the suggested control strategies can reduce the true prevalence to some extent but none of them can eliminate the infection from the herd. It has been debated previously that culling low or high shedders or both is not always justifiable^22,23,36^. Our results showed that culling all the red animals immediately (aggressive culling) is a straightforward solution to reduce prevalence (see Fig. 3) and reduce the importance of two key transmission routes: dam-to-daughter and colostrum/milk (see Fig. 4). However, as may be expected, the efficacy of this test and cull strategy also depended on the testing frequencies.

Previously, it was indicated that breaking or closing transmission routes can reduce the prevalence, but elimination has not yet been demonstrated in any field studies^20,37^. In our results, aggressive culling (control I) was not able to show elimination, despite reducing the number of shedders in both the short and long run (see Supplementary Tables S9, S10, and S11) because MAP persisted in the calf and heifer rearing populations (see Figs 4 and 5). This supports the finding of Nielsen and Toft’s study that the best action to reduce prevalence was to cull test-positive animals^18^. However, it has been indicated that aggressive culling (control I) is not feasible in reality, as it is economically unattractive to cull low shedding animals^20,38,39^. With this in mind, we explored three other control strategies; we found that culling dam and offspring (control III), in which animals leave the herd after completing their milking cycle, performed similarly to aggressive culling (control I) in some cases (see Fig. 3). In control III, daughters from red marked dams were removed from the herd, which reduced transmission in the calf and heifer rearing groups. For bi-annual and quarterly testing, culling dam and offspring (control III) showed very few transmission events in vertical (see Fig. 5d), calf-to-calf (see Supplementary Fig. S7), and heifer-to-heifer (see Fig. 6d) routes. Although a test-and-cull strategy alone is not sufficient to eliminate transmission, the knowledge of lactation, DIM, and disease status of the red animals could provide economically important decision support. However, this model does not yet support a full economic analysis of these control strategies. If a cow is diagnosed as clinically infected or high-risk, culling may be delayed because of pregnancy. In such a situation, farmers may use other control strategies based on risk. Figure 3c shows that even using quarterly testing, it is not possible to reduce the true or observed herd prevalence to zero with these delayed culling control strategies. Among the control strategies, aggressive culling showed the best performance in reducing shedders but was not able to eliminate all shedders.

Global sensitivity analysis showed that, in the short term (i.e. 5 years after intervention), infectious adults were the most influential population group in the model. We varied the parameters by 25%, 50%, 75% and 95% of their base values but chose 50% of the range in order to avoid the unrealistic model behavior as there many heterogeneous processes involved in the three layers of the IBM. We also performed sensitivity analysis for true prevalence 10 and 15 years after the intervention. In the longer term, parameters like calf-to-calf and heifer-to-heifer infection routes were more influential. This is likely because, when control strategies are implemented for longer periods, elimination can only be possible when preventive measures are taken against both critical transmission routes: calf-to-calf and heifer-to-heifer. These preventive measures are not covered in the common suggestions for herd hygiene in the current model, which include a clean and dry maternity area protected from manure from other adult cattle, separating calves from dams immediately after birth, no use of colostrum from red animals, no use of pooled colostrum/milk to calves and minimal exposure of calves and heifers to the manure of mature cattle^40^.

The presented IBM differs in many ways from other existing models of MAP. Our model considered the risk-based culling of highly infectious cows and three testing frequencies to evaluate each transmission route, while other control measures were adopted in other studies^26,27^. Robin *et al*. found that their new ethanol vortex ELISA (EVELISA) was cost-effective and quarterly test-and-cull control was able to significantly reduce the prevalence, but collecting serum for this test would increase labour costs and may not be realistic^27^. We simulated the use of a milk-based ELISA, which has been adopted in Denmark’s national JD eradication program due to the ease of collecting milk samples^41^. Likewise, it considered four control strategies: ELISA, management, ELISA and management and faecal testing and management^22,23^. The authors showed that the prevalence could be reduced to zero when faecal testing and management control was run at least 50 years, which is too long for practical use in herd management. The SimHerd model developed by Kudahl, in contrast, requires faecal culture confirmation of ELISA-positive cows before culling. This culture confirmation of ELISA positive cows would increase specificity but decrease sensitivity compared to our model’s milk ELISA, where we relied on repeated testing to find the most infectious animal^20^. A recent mechanistic bioeconomic model showed that MAP can be eradicated, but it is economically unattractive^41^. The model was parameterised specifically for Danish conditions by using a dataset obtained from the Danish Cattle Database hosted by SEGES (www.seges.dk/en). However, our simulation results did not show any eradication. There could be two reasons for this: first, our model did not consider any additional preventive measures and second, the model was parameterised from the Regional Dairy Quality Management Alliance (RDQMA) dataset^42^. Nevertheless, our main goal was not to eradicate disease, but to consider relevant information related to each individual which is needed to break the important transmission routes in endemic conditions.

A critical aspect for MAP detection is testing methods, as no testing method is perfect and also most testing results are variable in different countries based on different farm management strategies. In this paper, we used ELISA testing results categorised by epidemiological groups and age susceptibility, but the results could be improved by using faecal culture or PCR test after a positive ELISA. However, a long waiting period (i.e. typically 4 to 8 weeks) for the faecal-culture results may trigger the spread of the infection^22^. However, farmers can implement combination testing with higher frequency, but that may not be cost-effective^41^. Although the model helps our thinking, it is always a relatively simple reflection of reality. However, our IBM is adaptive in nature and can be expanded to include more details regarding specific control measures, MAP dose, environmental contamination, and economic impacts of control.

Our model calibration exercise (described in Supplementary subsection B) aimed to parameterise the model for three different hygiene scenarios. The parameters related to adult herd and hygiene coefficients were calibrated before implementing any of the control strategies. Nevertheless, some parameters like *β*_*A*_ *β*_*a*_, *β*_*c*_ and *β*_*m*_ were kept fixed during this calibration method, as these parameters have not been seen to be varied in certain ranges in other studies^15,17–21,28^. One reason is that the calf rearing and heifer rearing loops are under separate field and management facilities where they have the overall impact of the hygiene as an indirect component, but not as a direct component. Similar simplification of an indirect component of transmission is considered in a dynamic model of bovine tuberculosis spread in Great Britain^43^. In future, more experiments will be needed for calf and heifer rearing housing parameters, especially in US scenarios where these facilities are heterogeneous in terms of management.

MAP is endemic in the bovine population in the US and can cause a major degradation of animal welfare. The idea of the best-suited model can only be validated when we can obtain data from the majority of US dairy herds. The findings of this study may be suitable for US scenarios, but care must be taken when translating these results for other countries. Control activities are not uniformly coordinated nationally and internationally due to the variation in different farm management policies and government programs. As with all models, our model is limited by its simplifications. This model allowed for three hygiene conditions by adjusting model parameters, but in reality, these hygiene conditions are the result of discrete hygiene and preventive measures adopted by farmers on a day-to-day basis. Insufficient data were available to explicitly model the effects of such measures on the probability of transmission, especially transmission via colostrum/milk. It is worth mentioning that the results presented here depend on the assumptions of the model and parameters.

In summary, IBMs can be used to explain infectious disease transmission routes and persistence mechanisms to test hypotheses that would be difficult to test experimentally. An IBM can be fed with real herd data to support important decisions such as which control strategy to implement and when to cull and can calculate the future consequences of not culling high-risk animals. Our current IBM shows the contribution of six transmission routes of MAP (adult-to-adult, dam-to-daughter–horizontal, dam-to-daughter-vertical, colostrum/milk, calf-to-calf, and heifer-to-heifer) in different hygiene management conditions (moderate, low, and poor). This paper contributes to the understanding of MAP infection dynamics and control in three different ways. Firstly, it is unlikely that the suggested risk-based culling strategies here can eliminate MAP from an endemic herd due to disease persistence in both calf and heifer rearing housing, although they can reduce the prevalence significantly in both the short and long run. Secondly, dam-to-daughter horizontal transmission and transmission via colostrum/milk are the two primary routes of infection for MAP, but other transmission routes are critically responsible for the MAP persistence. Thirdly, only serial MAP testing can considerably reduce the number of infection events. In conclusion, controlling infectious disease in real life requires information about the truly infected individuals on many scales. For providing this information, an IBM like the one proposed here can play an invaluable role as an applied tool for other infectious diseases.

## Electronic supplementary material

eSupplementary Information

## References

[1] NAHMS. D 2007–Johne’s Disease on U.S. Dairies, 1991–2007. National Animal Health Monitoring System, Fort Collins, CO (2007).

[2] Ott SL, Wells SJ, Wagner BA (1999). Herd-level economic losses associated with Johne’s disease on US dairy operations. Prev. Vet. Med..

[3] Smith RL (2009). A longitudinal study on the impact of Johne’s disease status on milk production in individual cows. J. Dairy Sci..

[4] VanLeeuwen JA (2010). Associations between reproductive performance and seropositivity for bovine leukemia virus, bovine viral-diarrhea virus, Mycobacterium avium subspecies paratuberculosis, and Neospora caninum in Canadian dairy cows. Prev. Vet. Med..

[5] Smith RL (2010). Effect of Johne’s disease status on reproduction and culling in dairy cattle. J. Dairy Sci..

[6] Kudahl AB, Nielsen SS (2009). Effect of paratuberculosis on slaughter weight and slaughter value of dairy cows. J. Dairy Sci..

[7] Cho J (2013). Cost-effective control strategies for Johne’s disease in dairy herds. Can. J. Agric. Econ..

[8] Coussens PM (2001). Mycobacterium paratuberculosis and the bovine immune system. Anim. Heal. Res. Rev..

[9] Chacon O, Bermudez LE, Barletta RG (2004). Johne’s Disease, Inflammatory Bowel Disease, and Mycobacterium paratuberculosis. Annu. Rev. Microbiol..

[10] Windsor PA, Whittington RJ (2010). Evidence for age susceptibility of cattle to Johne’s disease. Vet. J..

[11] van Roermund HJW, Bakker D, Willemsen PTJ, de Jong MCM (2007). Horizontal transmission of Mycobacterium avium subsp. paratuberculosis in cattle in an experimental setting: Calves can transmit the infection to other calves. Vet. Microbiol..

[12] Whittington RJ, Begg DJ, de Silva K, Plain KM, Purdie AC (2012). Comparative immunological and microbiological aspects of paratuberculosis as a model mycobacterial infection. Vet. Immunol. Immunopathol..

[13] SDBbio. Review of On-Farm Bovine Johne’s Disease Management Strategies for Victorian Cattle Herds, Final project report 2014. (http://www.vff.org.au/) Accessed on 12 December 2015.

[14] Mortier RAR, Barkema HW, De Buck J (2015). Susceptibility to and diagnosis of Mycobacterium avium subspecies paratuberculosis infection in dairy calves: A review. Prev. Vet. Med..

[15] Smith RL, Schukken YH, Gröhn YT (2015). A new compartmental model of Mycobacterium avium subsp. paratuberculosis infection dynamics in cattle. Prev. Vet. Med..

[16] Schukken YH (2015). Longitudinal data collection of Mycobacterium avium subspecies Paratuberculosis infections in dairy herds: the value of precise field data. Vet. Res..

[17] Marcé C, Ezanno P, Seegers H, Pfeiffer D, Fourichon C (2011). Predicting fadeout versus persistence of paratuberculosis in a dairy cattle herd for management and control purposes: A modelling study. Vet. Res..

[18] Nielsen SS, Toft N (2011). Effect of management practices on paratuberculosis prevalence in Danish dairy herds. J. Dairy Sci..

[19] Dorshorst NC, Collins MT, Lombard JE (2006). Decision analysis model for paratuberculosis control in commercial dairy herds. Prev. Vet. Med..

[20] Kudahl AB, Østergaard S, Sørensen JT, Nielsen SS (2007). A stochastic model simulating paratuberculosis in a dairy herd. Prev. Vet. Med..

[21] Mitchell RM (2008). Simulation modeling to evaluate the persistence of Mycobacterium avium subsp. paratuberculosis (MAP) on commercial dairy farms in the United States. Prev. Vet. Med..

[22] Lu Z (2008). The importance of culling in Johne’s disease control. J. Theor. Biol..

[23] Lu Z, Schukken YH, Smith RL, Grohn YT (2010). Stochastic simulations of a multi-group compartmental model for Johne’s disease on US dairy herds with test-based culling intervention. J. Theor. Biol..

[24] Collins MT, Gardner IA, Garry FB, Roussel AJ, Wells SJ (2006). Consensus recommendations on diagnostic testing for the detection of paratuberculosis in cattle in the United States. J. Am. Vet. Med. Assoc..

[25] Verdugo C, Toft N, Nielsen SS (2015). Within- and between-herd prevalence variation of Mycobacterium avium subsp. paratuberculosis infection among control programme herds in Denmark (2011–2013). Prev. Vet. Med..

[26] Davidson RS (2012). Accounting for uncertainty in model-based prevalence estimation: paratuberculosis control in dairy herds. BMC Vet. Res..

[27] Robins J (2015). Agent-based model for Johne’s disease dynamics in a dairy herd. Vet. Res..

[28] Al-Mamun MA, Smith RL, Schukken YH, Gröhn YT (2016). Modeling of Mycobacterium avium subsp. paratuberculosis dynamics in a dairy herd: An individual based approach. J. Theor. Biol..

[29] Grimm V (2006). A standard protocol for describing individual-based and agent-based models. Ecol. Modell..

[30] USDA. Part I: Reference of Dairy Cattle Health and Management Practices in the United States (2007).

[31] Kudahl AB, Nielsen SS, Østergaard S (2008). Economy, efficacy, and feasibility of a risk-based control program against paratuberculosis. J. Dairy Sci..

[32] Saltelli, A., Chan, K. & Scott, E. M. editors. Sensitivity analysis. J. Wiley & Sons, New York (2000).

[33] Dairy Management-UW Extension. 2015. http://dysci.wisc.edu/research/dairy-management/ (Accessed on 10^th^ December, 2015).

[34] Hertl JA, Schukken YH, Welcome FL, Tauer LW, Gröhn YT (2014). Effects of pathogen-specific clinical mastitis on probability of conception in Holstein dairy cows. J. Dairy Sci..

[35] Hertl JA (2010). Effects of clinical mastitis caused by gram-positive and gram-negative bacteria and other organisms on the probability of conception in New York State Holstein dairy cows. J. Dairy Sci..

[36] Mitchell RM, Whitlock RH, Gröhn YT, Schukken YH (2015). Back to the real world: Connecting models with data. Prev. Vet. Med..

[37] Kudahl A, Nielsen SS, Sørensen JT (2004). Relationship between antibodies against Mycobacterium avium subsp. paratuberculosis in milk and shape of lactation curves. Prev. Vet. Med..

[38] Groenendaal H (2002). A simulation of Johne’s disease control. Prev. Vet. Med..

[39] Groenendaal H, Nielen M, Hesselink JW (2003). Development of the Dutch Johne’s disease control program supported by a simulation model. Prev. Vet. Med..

[40] Espejo LA, Godden S, Hartmann WL, Wells SJ (2012). Reduction in incidence of Johne’s disease associated with implementation of a disease control program in Minnesota demonstration herds. J. Dairy Sci..

[41] Kirkeby C (2016). Simulating the Epidemiological and Economic Impact of Paratuberculosis Control Actions in Dairy Cattle. Front. Vet. Sci..

[42] Pradhan AK (2009). Dynamics of endemic infectious diseases of animal and human importance on three dairy herds in the northeastern United States. J. Dairy Sci..

[43] Brooks-Pollock E, Roberts GO, Keeling MJ (2014). A dynamic model of bovine tuberculosis spread and control in Great Britain. Nature.

