# Supplementary material for: Use of an Individual-based Model to Control Transmission Pathways of Mycobacterium avium Subsp. paratuberculosis Infection in Cattle Herds
Source: Sci Rep. 2017 Sep 19;7:11845. doi: 10.1038/s41598-017-12078-z (PMC5605505; doi:10.1038/s41598-017-12078-z)
Supplement: Supplementary file 1 — eSupplementary Information [file 41598_2017_12078_MOESM1_ESM.doc]

**eSupplementary Information**

**Use of an Individual-based Model to Control Transmission Pathways of *Mycobacterium avium* Subsp. *paratuberculosis* Infection in Cattle Herds**

M.A. Al-Mamun, R. L. Smith, Y.H. Schukken, Y.T. Gröhn.

**A. Model description**

This supplementary section provides details of the state variables and presents the pseudo-code of the used functions: lactation status, birth, survival and additional culling, milking herd loop, calf rearing loop, heifer rearing loop and adult infection and progression.

Supplementary Table S1: List of state variables (structured in groups) used in the individual-based model (IBM) for Johne’s disease.

| **Cow** | **Lactation status** | **Infection status** | **ELISA Testing** | **Time** |
| --- | --- | --- | --- | --- |
| Adults  Calves  Heifers | Voluntary waiting period (VWP)  Inseminations  Pregnancy  Dry period (DP) | Susceptible  Latent  Low shedder  High shedder | Green cow  Yellow cow  Red cow | Animal age  Simulation steps |


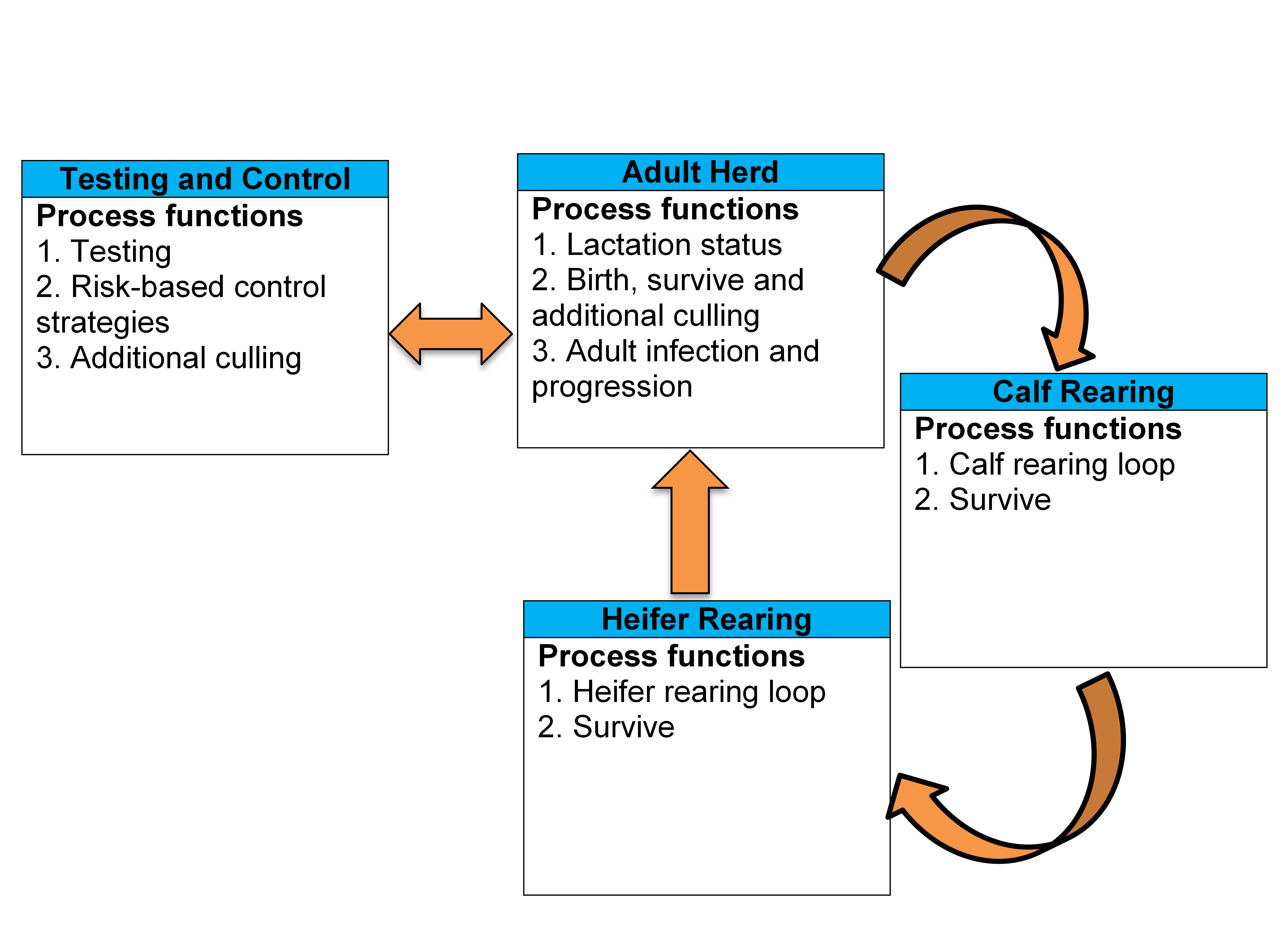


Supplementary Figure S1. Functions used in process overview and scheduling of the proposed individual-based model.

Description of Regional Dairy Quality Management Alliance (RDQMA) Dataset: The Regional Dairy Quality Management Alliance has collected data from 3 commercial dairy herds (herein, A, B, and C), consisting primarily of Holsteins, in the northeast United States (New York, Pennsylvania, and Vermont) between 2004 and 2010 [1]. Herds were visited quarterly to obtain serum samples from all adult animals (those that had calved at least once) and biannually to obtain fecal samples from all adult animals, and all monthly production and health records were obtained through the Dairy Herd Improvement Association (DHIA).

Supplementary Table S2. Basic information about a dairy animal used to parameterize the individual based model of a US dairy herd.

| **Description** | **Value in the model** | **References** |
| --- | --- | --- |
| Newborn | 1 day | [2] |
| Calves | 2-60 days | [2] |
| Heifers | 61-720 days | [2] |
| Adults | >720 days | [2] |
| Voluntary waiting period | 60 days | Calculated from RDQMA, [2] |
| Chance of producing female calf | 0.5 | [3] |
| Length of pregnancy | 280 days | [1] |
| Maximum length of insemination period before culling | 225 days | Calculated from RDQMA, [1] |
| Daily probability of adults death/sold (*µ_a_*) | 0.00055 | Calculated from RDQMA, [1] |
| Daily probability of calves death (*µ_c_*) | 0.000167 | Calculated from RDQMA, [1] |
| Daily probability of heifer death (*µ_h_*) | 0.0000278 | Calculated from RDQMA, [1] |
| Annual culling (%) | 30-40 | Calculated from RDQMA, [1] |
| Pregnancy percentage (annual) (%) | 70 | Calculated from RDQMA, [2, 4] |

Abbreviations: RDQMA, Regional Dairy Quality Management Alliance.

Sub-model: Lactation status

| Function (Lactation status) |
| --- |
| Precondition: Initial states of the model are set with 200 agents with random age, and have initialized the different parities  1 while there are any adults, agents do:  2 check the status  3 if voluntary waiting period, then  4 wait 60 days; increase age by 1  5 elseif inseminations  6 check the probability of insemination  7 if insemination is successful, then  8 flagged as pregnant  9 else  10 wait for next day  11 end if  12 elseif pregnant  13 check the pregnancy date (preg_d) == 280  14 if preg_d == 280, then  15 calving and stay with mother for one day  16 check infection probability by adults (horizontally and vertically)  17 if rand_num < vert_inf (Vhm, Vy1, Vy2)  18 set the inf_path flag=1, infected vertically  19 set inf_stat=1, infected calf  20 elseif rand_num < Inf_adult_to_calf(calculating from  equation 2)  21 set the inf_path flag=2, infected horizontally  22 set inf_stat=1, infected calf  23 end if  24 else  25 increase age by 1  26 end if  27 end if  28 end while... |
|  |

**Sub-model: birth, survival** and additional culling

| Function (birth, survival and additional culling) |
| --- |
| Precondition: initial states of the model are set with 200 agents with random age, and have initialized the different parities  1 check few condition  2 adult_survival:  3 if rand_num < 0.00055, then  4 flagged dead  5 else  6 alive  7 end if  8 calf_survival:  9 if rand_num < 0.000167, then  10 flagged dead  11 else  12 alive  13 end if  14 heifer_survival:  15 if rand_num < 0.0000278, then  16 flagged dead  17 else  18 alive  19 end if  20 birth: if any new birth event occurs  21 count=count+1;  22 additional culling  23 while red_animal is determined by control strategies  24 cull it based on criteria  25 end while…. |

Sub-model: Milking herd loop

| Function (Milking herd loop) |
| --- |
| Precondition: initial states of the model are set with 200 agents with random age, and have initialized the different parities  1 check the who_flag of the animal  2 if who_flag == 1, then  3 keep the animal in group 1: adult group  4 elseif who_flag == 2, then  5 keep the animal in group 2: maternity pen (first day after birth)  6 elesif who_flag == 3, then  7 keep the animal in group 3: calf group  8 elseif who_flag == 4, then  9 keep the animal in group 4: heifer group  10 end if  11 check probability of adult infection (@function: adult infection and progression)  12 check probability of adult-to-calf infection  13 if rand_num < Inf_adult_to_calf (calculating from equation 2)  14 set inf_path flag=2, infected horizontally  15 set inf_stat=1  16 else  17 set inf_stat=0  18 end if |

Sub-model: calf rearing loop

| Function (calf rearing loop) |
| --- |
| Precondition: initial states of the model are set with 200 agents with random age, and have initialized the different parities  1 check the infection status  2 if inf_stat == 1  3 she was infected by either vertically or horizontally and check inf_path flag  4 elseif inf_stat == 0  5 check probability of infection from colostrum/milk and from infected calves  6 if rand_num < beta_m, then  7 set the inf_path flag=3, infected by colostrum/milk  8 set inf_stat=1, infected heifer  9 elseif rand_num < Inf_calf_to_calf (calculating from equation 3)  10 set inf_path flag=4, infected by calves  11 else  12 do nothing and continue normal calf operation  13 end if  14 end if |

Sub-model: heifer rearing loop

| Function (heifer rearing loop) |
| --- |
| Precondition: initial states of the model are set with 200 agents with random age, and have initialized the different parities  1 check the infection status  2 if inf_stat == 1  3 animal was infected from calf rearing see inf_path flag  4 set inf_stat=2, infected heifer  5 elseif inf_stat == 0, susceptible heifer  6 set inf_stat=3 , susceptible heifer  7 check the probability of infection from other heifers  8 elseif rand_num < Inf_heifer_to_heifer (calculating from equation 4)  9 set inf_path flag=5, infected by heifers  10 set inf_stat= 2, infected heifer  11 else  12 do nothing and continue normal heifer operation  13 end if  14 end if  15 if heifer_age > 720 days, then  16 change who_flag = 1  17 set preg_d == 280  18 else  19 increase age by 1 and check the probability of infection by infected heifers  20 end if |

**Sub-model: adult infection and** progression

| Function (adult infection and progression) |
| --- |
| Precondition: initial states of the model are set with 200 agents with random age, and have initialized the different parities  1 For adult loop  2 if the inf_stat == 2, then  3 set inf_stat = 4, latent animal, check progression probability  4 if rand_num < 0.0013, then  5 set inf_stat =5, it is low shedder  6 else  7 keep the same status  8 end if  9 elseif inf_stat == 3, then  10 they are susceptible adults, check probability of getting infected by adults  11 if rand_num < Inf_adult_adult (calculating from equation 1)  12 set inf_stat = 4, now infected by adults  13 set inf_path = 5  14 else  15 normal operation and check the infection probability next day  16 end if  17 elseif inf_stat == 5, then  18 it is a low shedder and check the probability of progress to high shedders  19 if rand_num < 0.00077, then  20 set inf_stat = 6, it is now high shedder  21 else  22 normal operation and check the progression probability  23 end if  24 end if |


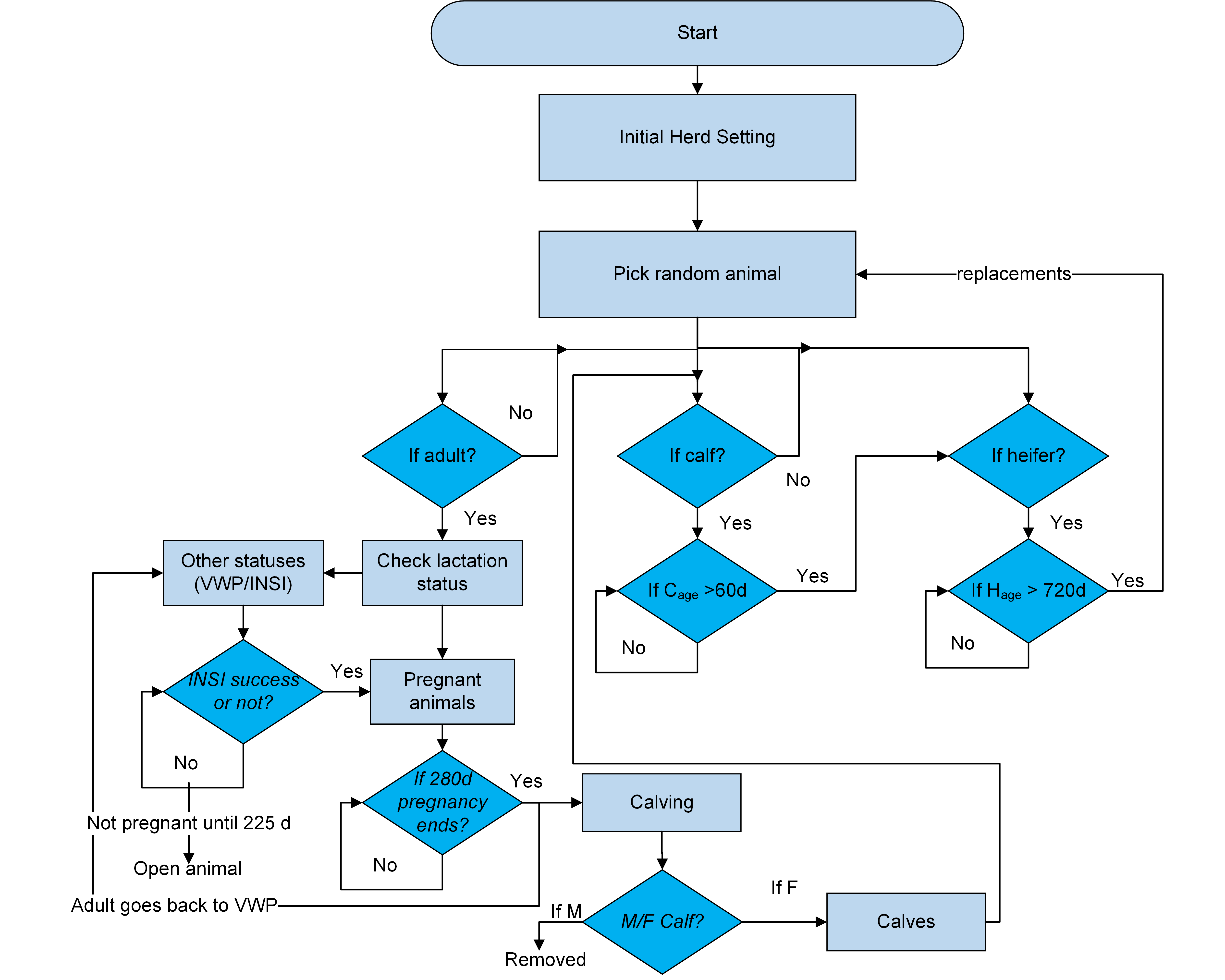


Supplementary Figure S2. Flow chart of daily event decisions for adults, calves and heifers in the milking herd. Abbreviations: VWP-voluntary waiting period, INSI-insemination, d-day, M-male, F-female, C_age_-calf’s age, H_age_-heifer’s age.

Supplementary Table S3: Stratified values for sensitivities and specificities of milk antibody enzyme-linked immunosorbent assay (ELISA) test based on days in milk and parities.

|  | **Sensitivity** | | | |
| --- | --- | --- | --- | --- |
| **Infection Group (parity)** | **DIM**  **(7-105d)** | **DIM**  **(106-203d)** | **DIM**  **(204-300d)** | **DIM**  **(>301d)** |
| Uninfected/Susceptible (1)^*^ | - | - | - | - |
| Latent (1) | 0.10 | 0.10 | 0.10 | 0.10 |
| Low shedders (1) | 0.50 | 0.60 | 0.65 | 0.70 |
| High shedders (1) | 0.80 | 0.80 | 0.80 | 0.80 |
| Uninfected/Susceptible (>1)^**^ | - | - | - | - |
| Latent (>1) | 0.10 | 0.10 | 0.10 | 0.10 |
| Low shedders (>1) | 0.60 | 0.70 | 0.75 | 0.75 |
| High shedders (>1) | 0.80 | 0.80 | 0.80 | 0.80 |

Abbreviations: DIM, days in milk; d, days.

*Specificity: 0.99, **Specificity: 0.96.

**B. Parameter values during model building and simulation**

This supplementary section provides the detailed parameter values used during model building and simulation. It also provides the model calibration method used in this paper.

Supplementary Table S4: List of parameters used during the model simulation.

| **Symbols** | **Description** | **Value in the model** | **References** |
| --- | --- | --- | --- |
| X_A_:H:Y_1_:Y_2_ | Initial ratio of susceptible to latent to low shedders to high shedders in the adult herd | 0.9:0.04:0.04:0.02 | [5] |
| *h_1_* | Disease progression rate from latent (H) to low shedding (Y_1_) (daily probability) | 0.0013 | [6] |
| *y_1_* | Disease progression rate from low shedding (Y_1_) to high shedding (Y_2_) (daily probability) | 0.00077 | [6] |
| *V_h_* | proportion of calves from latent animals infected at birth | 0.15 | [7] |
| *V_y1_* | proportion of calves from low-shedding animals infected at birth | 0.15 | [8] |
| *V_y2_* | proportion of calves from high-shedding animals infected at birth | 0.17 | [8] |
| $\beta_{A}$ | Adult-to-adult transmission coefficient | 0.05 | Calibrated in the model |
| $\beta_{a}$ | Adult-to-calf transmission coefficient | 0.383 | Calibrated in the model |
| $\beta_{c}$ | Calf-to-calf transmission coefficient | 0.0025 | [9] |
| $\beta_{m}$ | Adult-to-calf transmission via colostrum (daily probability) | 0.072 | Calibrated in the model |
| $\beta_{h}$ | Heifer-to-heifer transmission coefficient | 0.001 | Calibrated in the model |
| $\beta_{Y_{1}}$ | Transmission rate between low shedders (Y_1_) and susceptible (X_A_) | 2/year | Calibrated in the model |
| $\beta_{Y_{2}}$ | Transmission rate between high shedders (Y_2_) and susceptible (X_A_) | 20/year | Calibrated in the model |
| *Inf_adult-adult_* | Fecal-oral transmission (adult-adult) | Calculated by equation 1 | (Calculated) |
| *Inf_adult-calf_* | Fecal-oral transmission (adult-calf) | Calculated by equation 2 | (Calculated) |
| *Inf_calf-calf_* | Calf-to-calf transmission (daily probability) | Calculated by equation 3 | (Calculated) |
| *Inf_heifer-heifer_* | Heifer-to-heifer transmission (daily probability) | Calculated by equation 4 | (Calculated) |
| *G_1_* | Moderate hygiene coefficient | 0.924 | Calibrated in the model |
| *G_2_* | Low hygiene coefficient | 0.424 | Calibrated in the model |
| *G_3_* | Poor hygiene coefficient | 0.218 | Calibrated in the model |

Parameter Search Space and Model Calibration: The IBM was simulated for three hygiene scenarios, three testing frequencies, and four risk-based control strategies. Our first task was to fit the MAP transmission model to three specified ranges of annual prevalence which represent three hygiene scenarios. The first step was to define a range for each hygiene parameter (*G_1_,G_2_* and *G_3_*). As the main route of MAP transmission is the fecal-oral route, we assume that during different hygiene conditions, the transmission rate between low shedders and susceptibles () and transmission rate between high shedders and susceptibles ($\beta_{Y_{2}}$) would vary. We defined arbitrary values for these two rates for each hygiene scenario. First, 50,000 parameter combinations were generated by sampling from independent uniform distributions spanning the specified ranges. Second, we searched the parameter space by looking at the output as specified prevalence ranges suggested for three different hygiene-level herds. Third, we determined the range for each parameter for each hygiene simulation based on 50 best fits. Finally, we ran the simulation model for each hygiene scenario with the obtained parameter ranges. Supplementary Table S5 displays the parameter ranges for three hygiene herds. All the yearly parameters translated into probabilities (*P=1-exp (-rate)*).

Supplementary Table S5: The range of parameter values used in the parameter search and model calibration.

| **Parameters** | ***G*** | **Selected *G*** | *h_1_***per year** | **Selected** *h_1_* **per year** | $\beta_{Y_{1}}$ **per year** | **Selected per year** | **per year** | **Selected per year** |
| --- | --- | --- | --- | --- | --- | --- | --- | --- |
| **Moderate Hygiene** | (0.832,0.924) | (0.845,  0.900) | (0.001,0.002) | (0.001-0.002) | (2,4) | (2,3) | (6,12) | (6,8) |
| **Low Hygiene** | (0.382,0.424) | (0.398,  0.402) | 0.002,  0.003) | 0.002-0.003) | (4,6) | (4,6) | (12,16) | (12,13) |
| **Poor Hygiene** | (0.196,0.218) | (0.202,  0.212) | 0.002,  0.003) | 0.0025-0.003) | (6,8) | (6,7) | (16,20) | (16,28) |

Analysis of farm dataset: The dataset used to obtain information on reproduction had reproduction records on 90,271 observations (between 1 and 4 artificial insemination attempts per lactation) in 39,361 lactations in 20,328 cows in 5 New York herds, collected from 2003-2004 until July 2011 [10]. Three herds were in central New York State, 1 was in northern New York State, and 1 was in western New York State. The farms recorded information on milk production and milk electrical conductivity, parity, reproduction, diseases, calving, drying-off, and herd exit using the DairyComp305 herd management software (Valley Agricultural Software, Tulare, CA). The dataset used to obtain information on death and culling had information on 23,409 cows, collected from 2003-2004 until 2011 [11, 12]. We calculated calving interval, success of inseminations, annual pregnancy percentage estimates from the reproduction dataset and culling estimates from the culling dataset. The variables relating to milk yield, mastitis culture results, diseases, and reproduction necessary to conduct this study were exported to ASCII files from DairyComp305 herd management software (Valley Agricultural Software, Tulare, CA) and imported into SAS v.9.2 software [13].

Comparing the risk-based culling strategies with no control: The model was simulated with three hygiene conditions by implementing the suggested risk-based control strategies. We used ONEWAY ANOVA tests to compare the control strategies followed by a post-test Bonferroni to determine the significant control strategy.

**C. Evaluation of control efficacy on different scenarios**

This section provides the relevant results in terms of tables and figures to support the simulation results.

**
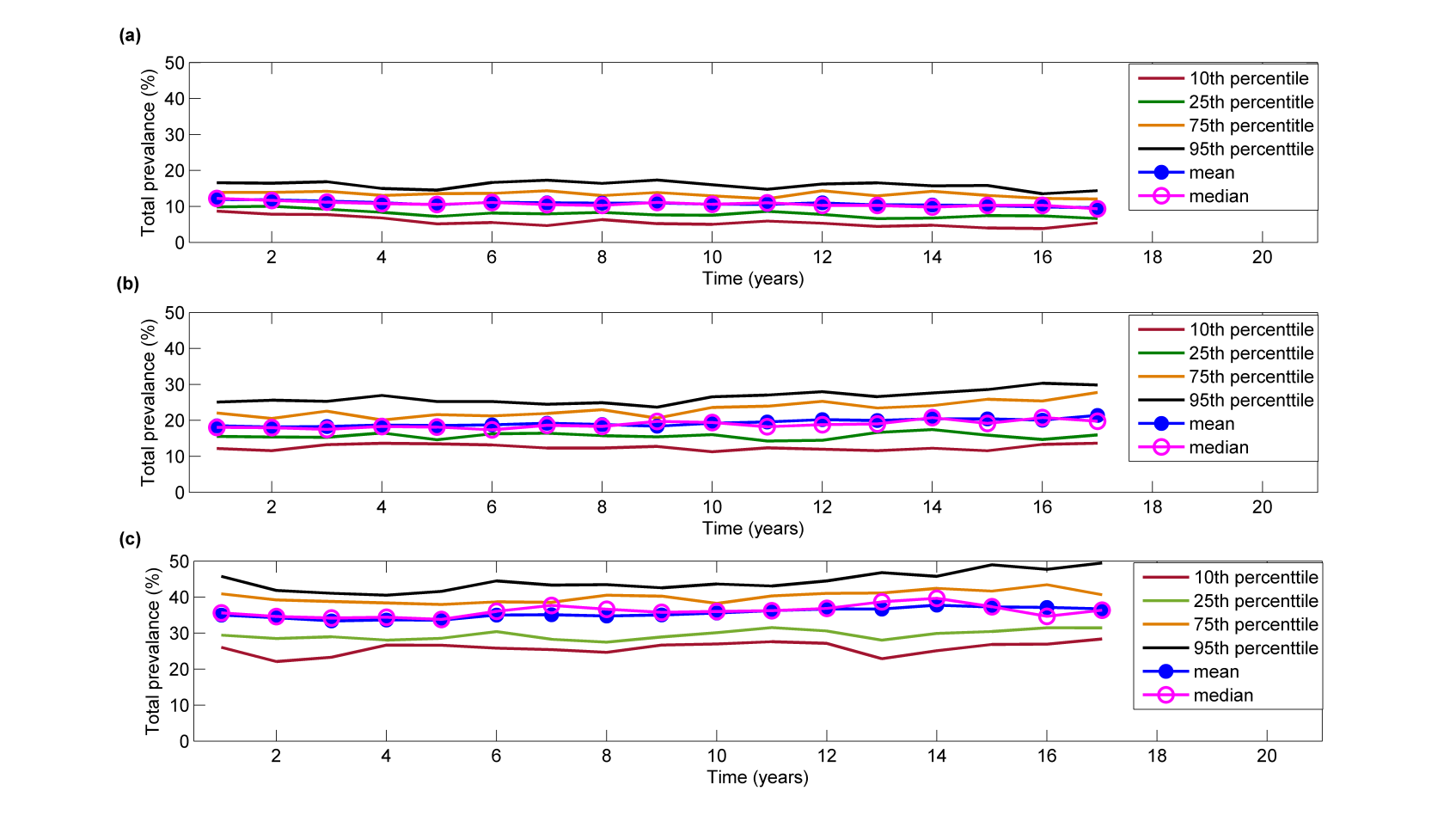
**

Supplementary Figure S3. Overall mean true prevalence of *Mycobacterium avium* subsp. *paratuberculosis* infection in simulated uncontrolled herds by hygiene scenarios. True prevalence was calculated as *(H+Y_1_+Y_2_)/N*, where H-latent, Y_1_-low shedder, and Y_2_-high shedder, and N is the total number of animals, (a) moderate hygiene herd, (b) low hygiene herd and (c) poor hygiene herd.

**
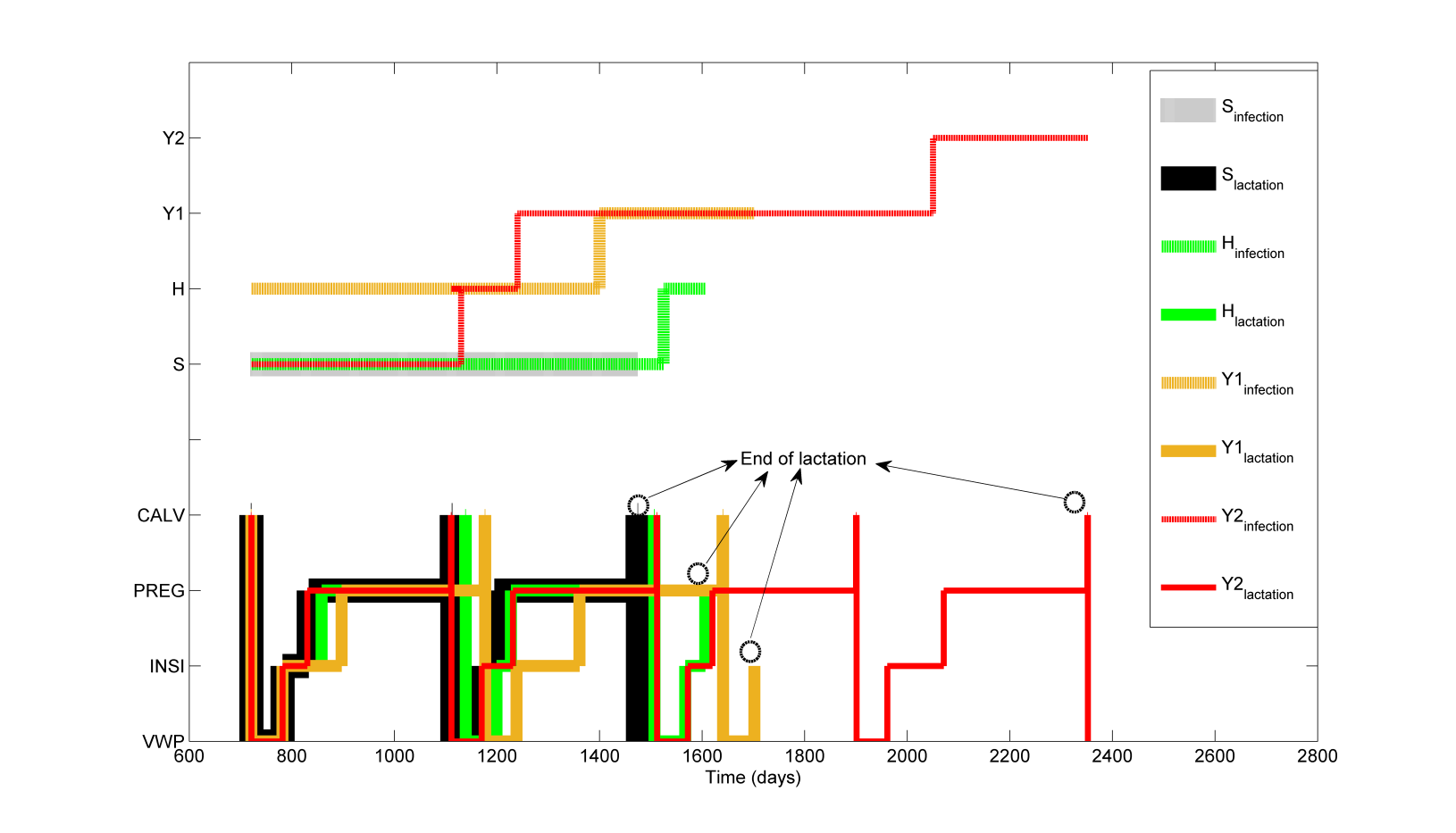
**

Supplementary Figure S4.The natural tracking of four animals in the adult herd by lactation stages and infection status. Abbreviations: VWP-voluntary waiting period, INSI-insemination, PREG-pregnant, CALV-calving and X_A_-susceptible, H-latent, Y_1_-low shedding, and Y_2_-high shedding animals. Colors represent individual animals.

Supplementary Table S6: The comparison of risk-based control strategies for each hygiene policy with three testing frequencies at fifth year while considering the significant level α < 0.05.

|  | **Annual Testing**  **(mean±stdev)** | **Biannual Testing**  **(mean±stdev)** | **Quarterly Testing (mean±stdev)** |
| --- | --- | --- | --- |
| **Moderate Hygiene** |  |  |  |
| **NC** | 10.7±2.7^a^ | 10.7±2.7^a^ | 10.7±2.7^a^ |
| **Con_I** | 6.0±2.1^b^ | 2.3±1.2^b^ | 1.4±1.0^b^ |
| **Con_II** | 9.7±3.1^c^ | 4.8±2.0^c^ | 2.5±1.3^c^ |
| **Con_III** | **6.4±2.5^b^** | 2.8±1.3^b^ | 1.6±0.9^b^ |
| **Con_IV** | **6.3±1.9^b^** | 2.7±1.3^b^ | 1.8±1.1^b^ |
| **Low Hygiene** |  |  |  |
| **NC** | 18.2±5.3^a^ | 18.2±5.3^a^ | 18.2±5.3^a^ |
| **Con_I** | 9.7±2.9^b^ | 3.9±1.6^b^ | 2.0±1.0^b^ |
| **Con_II** | 16.3±3.9^c^ | 8.1±2.7^c^ | 4.0±1.6^c^ |
| **Con_III** | **11.0±3.6^d^** | 4.0±1.9^b^ | 2.4±1.2^b^ |
| **Con_IV** | **11.4±2.9^d^** | 4.4±1.7^b^ | 2.4±1.3^b^ |
| **Poor Hygiene** |  |  |  |
| **NC** | 32.3±6.4^a^ | 32.3±6.4^a^ | 32.3±6.4^a^ |
| **Con_I** | 15.8±2.5^b^ | 5.5±1.9^b^ | 2.7±1.1^b^ |
| **Con_II** | 25.5±5.1^c^ | 12.8±3.2^c^ | 5.8±1.9^c^ |
| **Con_III** | **17.9**±**4.1^d^** | 6.6±2.6^b^ | 3.0±1.4^b^ |
| **Con_IV** | **18.1**±**3.67^d^** | 6.6±2.2^b^ | 3.3±1.3^b^ |

a,b,c,d: The same subscript indicates that the prevalences do not statistically differ from each other. Different subscripts indicate that the prevalences statistically differ from each other.

Supplementary Table S7: The comparison of risk-based control strategies for each hygiene policy with three testing frequencies at tenth year while considering the significant level α ≤ 0.05.

|  | **Annual Testing**  **(mean±stdev)** | **Biannual Testing**  **(mean±stdev)** | **Quarterly Testing (mean±stdev)** |
| --- | --- | --- | --- |
| **Moderate Hygiene** |  |  |  |
| **NC** | 10.7±2.8^a^ | 10.0±4.2^a^ | 10.0±4.24^a^ |
| **Con_I** | 3.7±1.6^b^ | 1.5±0.96^b^ | 1.0±0.63^b^ |
| **Con_II** | 7.2±3.2^c^ | 3.0±1.59^c^ | 1.6±0.91^b^ |
| **Con_III** | **4.1±2.0^b^** | 1.6±0.97^b^ | **0.9±0.77^c^** |
| **Con_IV** | **4.3±1.8^b^** | 1.6±1.1^b^ | **1.1±0.76^b^** |
| **Low Hygiene** |  |  |  |
| **NC** | 20.1±5.2^a^ | 20.1±5.2^a^ | 20.1±5.26^a^ |
| **Con_I** | 6.5±2.7^b^ | 1.9±1.2^b^ | **1.1±0.67^b^** |
| **Con_II** | 14.5±5.04^c^ | 5.2±2.6^c^ | **1.8±1.21^b^** |
| **Con_III** | **7.1±3.18^b^** | 2.3±1.34^b^ | **1.2±0.89^b^** |
| **Con_IV** | **7.2±2.72^b^** | 2.3±1.33^b^ | **1.4±0.89^b^** |
| **Poor Hygiene** |  |  |  |
| **NC** | 33.3±7.5^a^ | 33.3±7.54^a^ | 33.3±7.54^a^ |
| **Con_I** | 9.9±3.9^b^ | 2.3±1.12^b^ | **1.2±0.74^b^** |
| **Con_II** | 22.1±5.8^c^ | 7.6±2.87^c^ | **2.3±1.17^b^** |
| **Con_III** | **11.0±3.9^b^** | 2.7±1.63^b^ | **1.4±0.89^b^** |
| **Con_IV** | **10.3±3.8^b^** | 3.0±1.62^b^ | **1.6±0.87^b^** |

a,b,c: The same subscript indicates that the prevalences do not statistically differ from each other. Different subscripts indicate that the prevalences statistically differs from each other.

Supplementary Table S8: The comparison of risk-based control strategies for each hygiene policy with three testing frequencies at fifteenth year while considering the significant level α < 0.05.

|  | **Annual Testing**  **(mean±stdev)** | **Biannual Testing**  **(mean±stdev)** | **Quarterly Testing (mean±stdev)** |
| --- | --- | --- | --- |
| **Moderate Hygiene** |  |  |  |
| **NC** | 10.2±4.2^a^ | 10.2±4.2^a^ | 10.2±4.2^a^ |
| **Con_I** | 2.6±1.5^b^ | 1.5±0.9^b^ | 0.8±0.6^b^ |
| **Con_II** | 5.7±2.9^c^ | 2.6±1.6^c^ | 1.1±0.8^b^ |
| **Con_III** | 2.9±1.7^b^ | 1.4±0.9^b^ | 1.1±0.8^b^ |
| **Con_IV** | 3.0±1.6^b^ | 1.5±0.9^b^ | 1.1±0.8^b^ |
| **Low Hygiene** |  |  |  |
| **NC** | 22.4±7.3^a^ | 22.4±7.3^a^ | 22.4±7.3^a^ |
| **Con_I** | 4.8±2.5^b^ | 1.4±0.8^b^ | 1.0±0.7^b^ |
| **Con_II** | 13.4±5.2^c^ | 4.1±2.2^c^ | 1.6±1.0^b^ |
| **Con_III** | 5.0±2.6^b^ | 1.8±1.2^b^ | 1.1±0.7^b^ |
| **Con_IV** | 5.5±2.7^b^ | 2.0±1.3^b^ | 1.2±0.8^b^ |
| **Poor Hygiene** |  |  |  |
| **NC** | 34.6±8.8^a^ | 34.6±8.8^a^ | 34.6±8.8^a^ |
| **Con_I** | 6.8±3.4^b^ | 1.6±1.0^b^ | 1.2±0.7^b^ |
| **Con_II** | 19.5±6.7^c^ | 5.1±2.6^c^ | 1.7±0.9^b^ |
| **Con_III** | 7.4±3.5^b^ | 1.8±1.2^b^ | 1.1±0.8^b^ |
| **Con_IV** | 7.0±3.1^b^ | 2.0±1.2^b^ | 1.3±0.8^b^ |

a,b,c: The same subscript indicates that the prevalences do not statistically differ from each other. Different subscripts indicate that the prevalences statistically differ from each other.


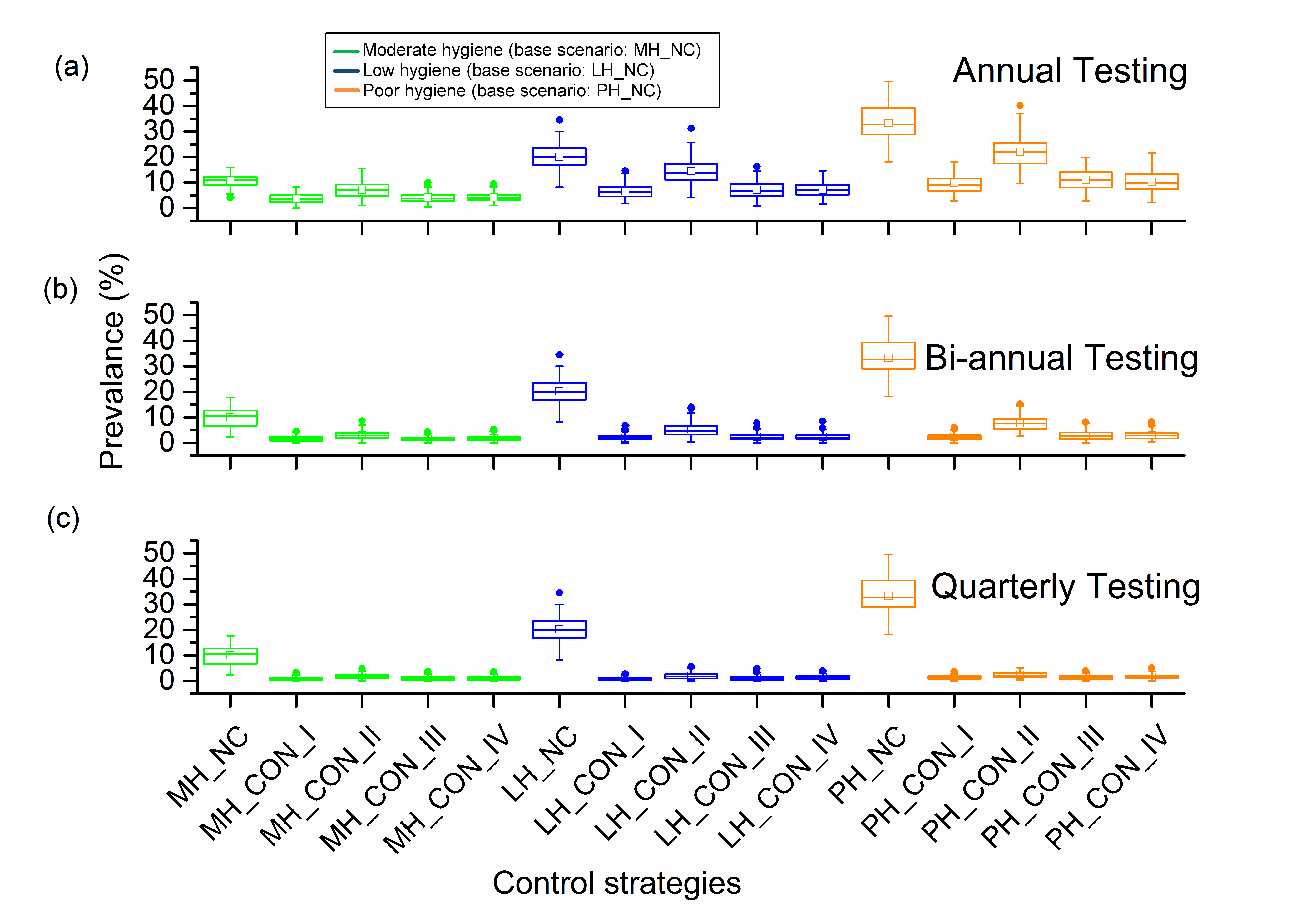


Supplementary Figure S5. Evaluation of control strategies in three hygiene conditions at tenth year for (a) annual testing, (b) bi-annual testing and (c) quarterly testing. In all box plots, the bottom and top end of the bars are minimum and maximum values respectively, the top of the box is the 75th percentile, the bottom of the box is the 25th percentile, and the horizontal line within the box is the median; outliers are presented as solid circles. Abbreviations: MH_NC: moderate hygiene-no control, MH_CON_I: moderate hygiene-control I, MH_CON_II: moderate hygiene-control II, MH_CON_III: moderate hygiene-control III, MH_CON_IV: moderate hygiene-control IV, LH_NC: low hygiene-no control, LH_CON_I: low hygiene-control I, LH_CON_II: low hygiene-control II, LH_CON_III: low hygiene-control III, LH_CON_IV: low hygiene-control IV, PH_NC: poor hygiene-no control, PH_CON_I: poor hygiene-control I, PH_CON_II: poor hygiene-control II, PH_CON_III: poor hygiene-control III, and PH_CON_IV: poor hygiene-control IV.

**
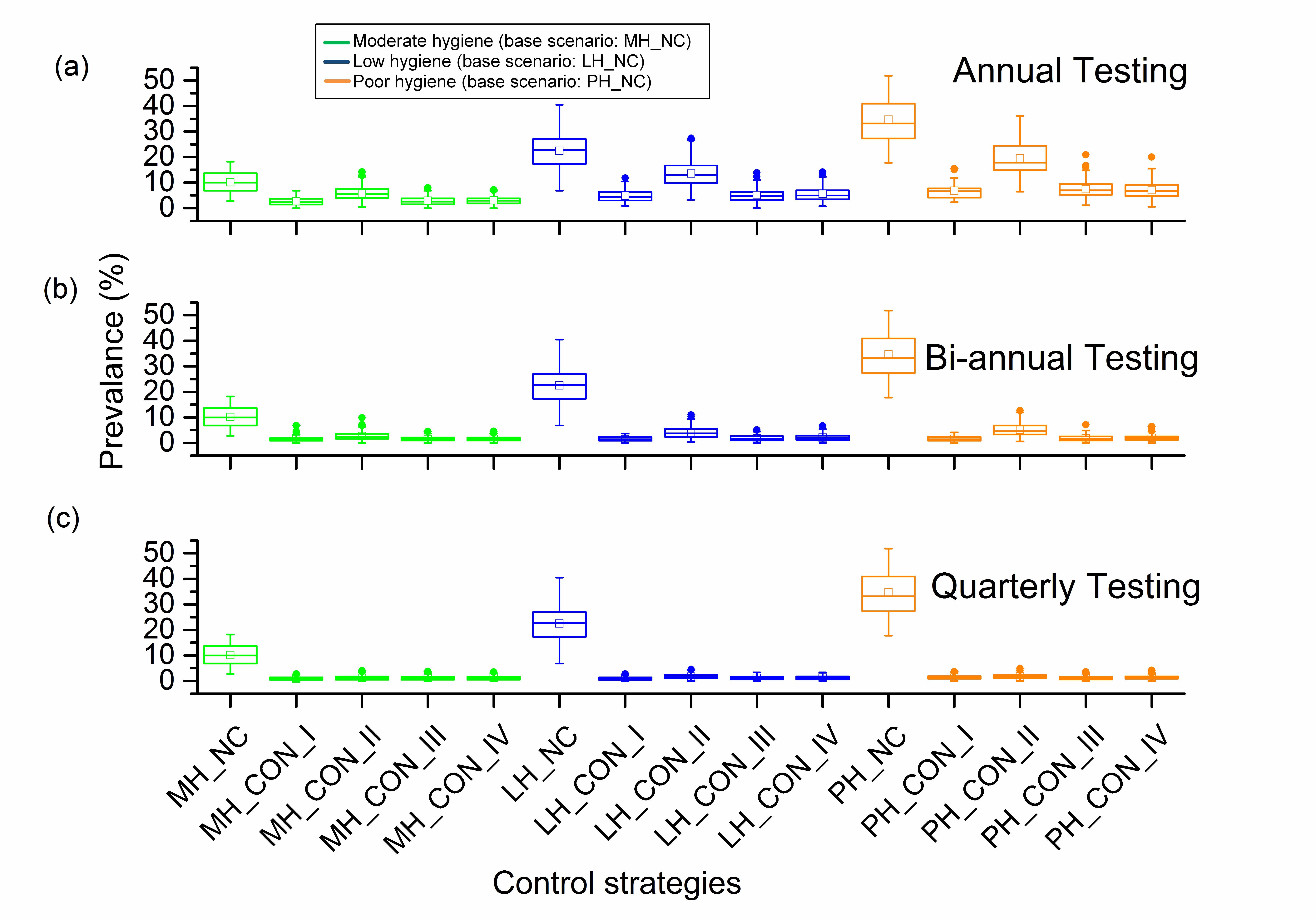
**

Supplementary Figure S6. Evaluation of control strategies in three hygiene conditions at fifteenth year for (a) annual testing, (b) bi-annual testing and (c) quarterly testing. In all box plots, the bottom and top end of the bars are minimum and maximum values respectively, the top of the box is the 75th percentile, the bottom of the box is the 25th percentile, and the horizontal line within the box is the median; outliers are presented as solid circles. Abbreviations: MH_NC: moderate hygiene-no control, MH_CON_I: moderate hygiene-control I, MH_CON_II: moderate hygiene-control II, MH_CON_III: moderate hygiene-control III, MH_CON_IV: moderate hygiene-control IV, LH_NC: low hygiene-no control, LH_CON_I: low hygiene-control I, LH_CON_II: low hygiene-control II, LH_CON_III: low hygiene-control III, LH_CON_IV: low hygiene-control IV, PH_NC: poor hygiene-no control, PH_CON_I: poor hygiene-control I, PH_CON_II: poor hygiene-control II, PH_CON_III: poor hygiene-control III, and PH_CON_IV: poor hygiene-control IV.


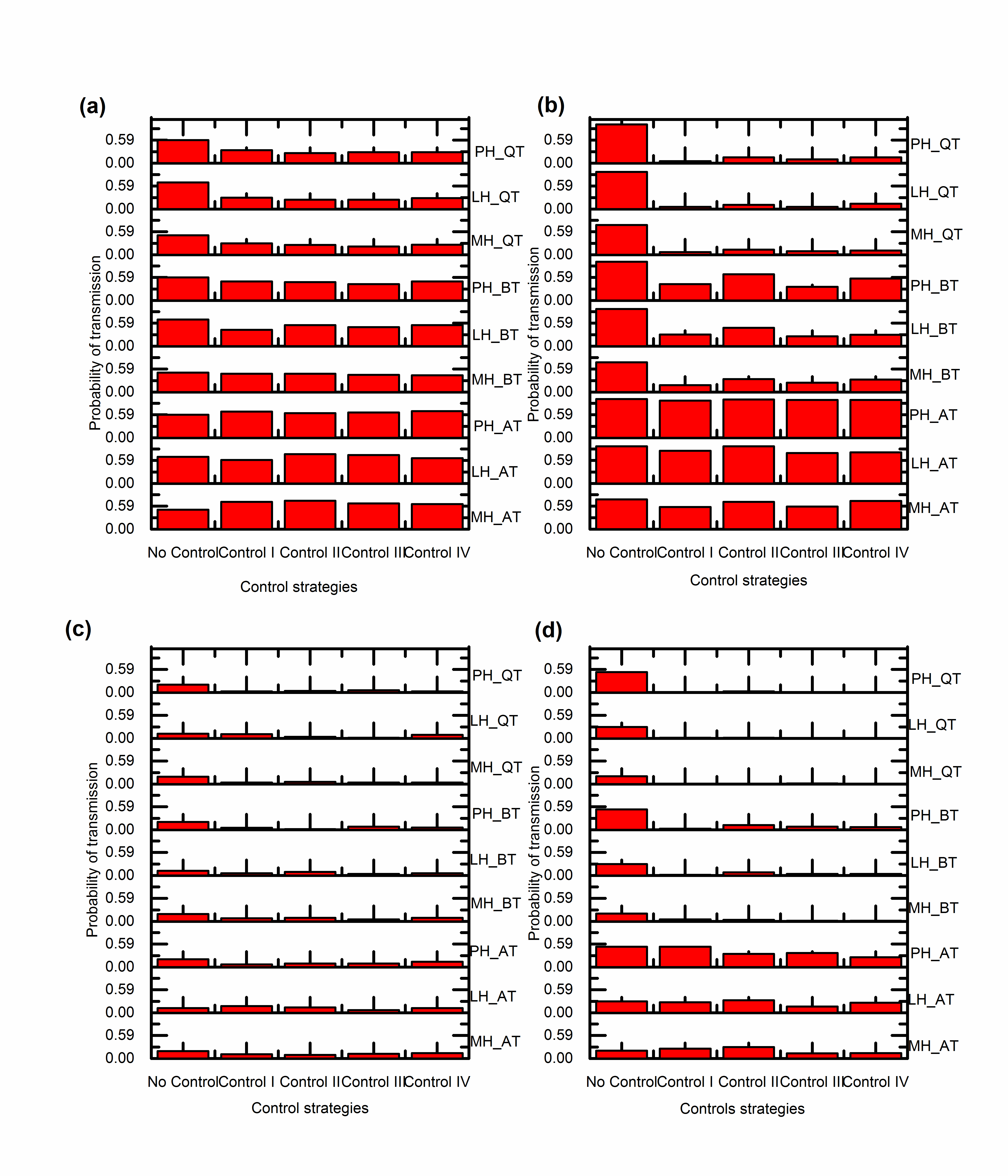


Supplementary Figure S7. Transmission probabilities by different routes after implementing risk-based control scenarios. The probability of transmission after implementing the control strategies for 9 different herd scenarios while considering (a) heifer-to-heifer transmission route, (b) vertical transmission route, (c) calf-to-calf transmission route and (d) adult-to-adult transmission route. Abbreviations: MH_AT: moderate hygiene-annual testing, LH_AT: low hygiene-annual testing, PH_AT: poor hygiene-annual testing, MH_BT: moderate hygiene-biannual testing, LH_BT: low hygiene-biannual testing, PH_BT: poor hygiene-biannual testing, MH_QT: moderate hygiene-quarter testing, LH_QT: low hygiene-quarter testing, and PH_QT: poor hygiene-quarter testing.

Supplementary Table S9. Prevalence and number of MAP shedders in endemically infected dairy herds after implementing risk based control strategies when tested annually. The percentages are presented as mean (95% Confidence Interval). True prevalence was calculated by using *(H+Y_1_+Y_2_)/N*; N is the total number of animals.

| **Moderate Hygiene** | **TP at 5^th^ year**  **Shedders (Y_1_+Y_2_)** | **TP at 10^th^ year**  **Shedders (Y_1_+Y_2_)** | **TP at 15^th^ year**  **Shedders (Y_1_+Y_2_)** |
| --- | --- | --- | --- |
| No control | 10.7(10.16-11.25) 5.35(.01-5.68) | 10.04(9.19-10.88) 5.09(4.66-5.51) | 10.15(9.32-10.98) 5.04(4.56-5.51) |
| Control I | **6.03(5.67-6.39) 2.9(2.68-3.13)** | **3.65(3.35-3.95) 1.73(1.55-1.9)** | **2.65(2.39-2.91) 1.22(1.07-1.37)** |
| Control II | 9.71(9.14-10.27)  5.00(4.2-4.81) | 7.2(6.62-7.77) 3.17(2.87-3.46) | 5.75(5.23-6.27) 2.67(2.39-2.95) |
| Control III | **6.41(5.96-6.85) 2.87(2.56-3.18)** | **4.15(3.8-4.5) 1.81(1.6-2.02)** | **2.91(2.6-3.22) 1.22(1.05-1.4)** |
| Control IV | 6.35(5.99-6.71) 2.86(2.62-3.09) | 4.28(3.95-4.61) 1.8(1.62-1.98) | 3.04(2.75-3.32) 1.23(1.09-1.38) |
| **Low Hygiene** |  | | |
| No control | 18.25(17.19-19.31) 9.03(8.54-9.52) | 20.13(19.08-1.17) 10.27(9.76-10.78) | 22.45(21-23.91) 11.15(10.38-1.93) |
| Control I | **9.7(9.18-10.22) 4.75(4.43-5.07)** | **6.55(6.05-7.04) 2.94(2.67-3.2)** | **4.81(4.36-5.26) 2.08(1.84-2.31)** |
| Control II | 16.4(15.7-17.09) 7.64(7.23-8.05) | 14.5(13.59-15.41) 6.6(6.12-7.08) | 13.5(12.57-14.43) 6.17(5.63-6.72) |
| Control III | **11.08(10.44-11.73)**  **4.9(4.58-5.22)** | **7.12(6.55-7.69) 2.84(2.57-3.12)** | **5.08(4.62-5.55) 1.97(1.77-2.18)** |
| Control IV | 11.44(10.92-11.96) 4.97(4.67-5.26) | 7.21(6.72-7.71) 2.83(2.57-3.08) | 5.5(5.01-5.99) 2.16(1.94-2.37) |
| **Poor Hygiene** |  | | |
| No control | 32.38(31.11-33.64) 16.15(15.43-16.86) | 33.33(31.83-4.82) 16.32(15.65-17.0) | 34.69(32.94-6.44) 17.32(16.34-8.29) |
| Control I | **15.88(15.43-16.33) 8.19(7.91-8.46)** | **9.86(9.15-10.56) 4.27(3.91-4.62)** | **6.84(6.24-7.45) 2.98(2.72-3.24)** |
| Control II | 25.59(24.66-26.51) 11.83(11.3-12.35) | 22.13(21.09-3.18) 9.83(9.26-10.4) | 19.5(18.29-20.72) 8.58(7.89-9.27) |
| Control III | **17.91(17.09-18.72) 6.5(5.85-7.14)** | **11.07(10.28-1.85)**  **3.7(3.27-4.12)** | **7.45(6.74-8.15) 2.26(1.98-2.54)** |
| Control IV | 18.18(17.51-18.84) 8.16(7.76-8.56) | 10.36(9.67-11.04) 4.39(4.06-4.73) | 7.07(6.51-7.64) 2.85(2.58-3.12) |

TP: Total prevalence, Y_1_: low shedders and Y_2_: high shedders

Supplementary Table S10. Prevalence and number of MAP shedders in endemically infected dairy herds after implementing risk based control strategies when tested biannually. The percentages are presented as mean (95% Confidence Interval). True prevalence was calculated by using *(H+Y_1_+Y_2_)/N*; N is the total number of animals.

| **Moderate Hygiene** | **TP at 5^th^ year**  **Shedders (Y_1_+Y_2_)** | **TP at 10^th^ year**  **Shedders (Y_1_+Y_2_)** | **TP at 15^th^ year**  **Shedders (Y_1_+Y_2_)** |
| --- | --- | --- | --- |
| No control | 10.7(10.16-11.25) 5.35(5.01-5.68) | 10.04(9.19-10.88) 5.09(4.66-5.51) | 10.15(9.32-10.98) 5.04(4.56-5.51) |
| Control I | **2.3(2.09-2.52) 0.89(0.77-1.01)** | **1.54(1.37-1.71) 0.52(0.42-0.62)** | **1.52(1.35-1.7) 0.58(0.49-0.68)** |
| Control II | 4.83(4.46-5.2) 2.02(1.84-2.19) | 3.05(2.76-3.34) 1.19(1.05-1.33) | 2.67(2.38-2.96) 1.04(0.91-1.17) |
| Control III | **2.83(2.6-3.07) 0.98(0.85-1.12)** | **1.61(1.44-1.79) 0.56(0.46-0.67)** | **1.5(1.33-1.66) 0.55(0.46-0.64)** |
| Control IV | 2.79(2.55-3.03) 0.93(0.81-1.04) | 1.68(1.49-1.88) 0.5(0.42-0.58) | 1.56(1.39-1.74) 0.51(0.42-0.6) |
| **Low Hygiene** |  | | |
| No control | 18.25(17.19-9.31) 9.03(8.54-9.52) | 20.13(19.08-1.17) 10.27(9.76-10.78) | 22.45(21.00-23.91) 11.15(10.38-11.93) |
| Control I | **3.95(3.67-4.24) 1.57(1.41-1.72)** | **1.98(1.76-2.21) 0.7(0.57-0.83)** | **1.5(1.34-1.65) 0.6(0.52-0.69)** |
| Control II | 8.14(7.65-8.62) 3.16(2.92-3.41) | 5.25(4.77-5.73) 2.08(1.85-2.32) | 4.12(3.72-4.51) 1.55(1.36-1.74) |
| Control III | **4.09(3.74-4.43) 1.41(1.25-1.57)** | **2.34(2.1-2.58) 0.78(0.66-0.89)** | **1.82(1.61-2.03) 0.55(0.46-0.64)** |
| Control IV | 4.46(4.16-4.76) 1.45(1.31-1.6) | 2.37(2.13-2.61) 0.77(0.65-0.89) | 2.07(1.84-2.3) 0.67(0.57-0.78) |
| **Poor Hygiene** |  | | |
| No control | 32.38(31.11-33.64) 16.15(15.43-16.86) | 33.33(31.83-34.82) 6.32(15.65-17.00) | 34.69(32.94-36.44) 17.32(16.34-18.29) |
| Control I | **5.54(5.18-5.89) 2.1(1.9-2.3)** | **2.36(2.16-2.57) 0.88(0.77-0.99)** | **1.65(1.47-1.83) 0.57 (0.47-0.67)** |
| Control II | 12.83(12.26-13.41) 5.09(4.79-5.39) | 7.63(7.11-8.15) 2.91(2.67-3.16) | 5.15(4.68-5.61) 1.82(1.6-2.03) |
| Control III | **6.64(6.18-7.11) 2.36(2.13-2.59)** | **2.78(2.49-3.08) 0.84(0.71-0.96)** | **1.84(1.62-2.07) 0.63(0.51-0.75)** |
| Control IV | 6.58(6.17-6.99) 2.27(2.1-2.44) | 3.01(2.72-3.3) 1(0.86-1.14) | 2.01(1.8-2.23) 0.63(0.52-0.73) |

TP: Total prevalence, Y_1_: low shedders and Y_2_: high shedders

Supplementary Table S11. Prevalence and number of MAP shedders in endemically infected dairy herds after implementing risk based control strategies when tested quarterly. The percentages are presented as mean (95% Confidence Interval). True prevalence was calculated by using *(H+Y_1_+Y_2_)/N*; N is the total number of animals.

| **Moderate Hygiene** | **TP at 5^th^ year**  **Shedders (Y_1_+Y_2_)** | **TP at 10^th^ year**  **Shedders (Y_1_+Y_2_)** | **TP at 15^th^ year**  **Shedders (Y_1_+Y_2_)** |
| --- | --- | --- | --- |
| No control | 10.71(10.16-11.25)  5.35(5.01-5.68) | 10.04(9.19-10.89)  5.09 (4.66-5.51) | 10.15(9.32-10.98)  5.04(4.56-5.51) |
| Control I | **1.43(1.26-1.6)**  **0.39(0.31-0.47)** | **1(0.89-1.12)**  **0.27(0.21-0.33)** | **0.88(0.77-1)**  **0.23(0.18-0.29)** |
| Control II | 2.58(2.34-2.82)  0.79(0.67-0.9) | 1.64(1.48-1.8)  0.43(0.35-0.51) | 1.15(1-1.3)  0.36(0.28-0.43) |
| Control III | **1.67(1.51-1.83)**  **0.36(0.28-0.45)** | **0.95(0.81-1.09) 0.18(0.12-0.23)** | **1.11(0.97-1.26)**  **0.25(0.18-0.31)** |
| Control IV | 1.85(1.65-2.04)  0.41(0.33-0.48) | 1.12(0.98-1.26) 0.24(0.19-0.3) | 1.16(1.03-1.3)  0.28(0.22-0.34) |
| **Low Hygiene** |  | | |
| No control | 18.25(17.19-19.31) 9.03(8.54-9.52) | 20.13(19.08-21.17) 10.27(9.76-10.78) | 22.45(21.00-23.91) 11.15(10.38-1.93) |
| Control I | **2.05(1.86-2.24)**  **0.47(0.39-0.55)** | **1.1(0.98-1.22)**  **0.27(0.21-0.33)** | **1.09(0.96-1.21)**  **0.25(0.19-0.31)** |
| Control II | 4.05(3.77-4.33)  1.3(1.16-1.45) | 1.89(1.67-2.11) 0.59(0.49-0.68) | 1.69(1.5-1.87)  0.47(0.38-0.56) |
| Control III | **2.38(2.17-2.58)**  **0.54(0.45-0.63)** | **1.21(1.04-1.37)**  **0.27(0.2-0.33)** | **1.2(1.07-1.33)**  **0.3(0.24-0.37)** |
| Control IV | 2.49(2.26-2.72)  0.62(0.51-0.73) | 1.41(1.25-1.57) 0.34(0.26-0.42) | 1.29(1.14-1.44)  0.33(0.26-0.4) |
| **Poor Hygiene** |  | | |
| No control | 32.38(31.11-33.64) 16.15(15.43-16.86) | 33.33(31.83-34.82) 16.32(15.65-17.00) | 34.69(32.94-36.44) 17.32(16.34-18.29) |
| Control I | **2.78(2.58-2.99)**  **0.82(0.71-0.92)** | **1.23(1.1-1.36)**  **0.29(0.22-0.35)** | **1.21(1.08-1.35)**  **0.33(0.27-0.4)** |
| Control II | 5.87(5.52-6.22)  2.0(1.82-2.18) | 2.39(2.18-2.6)  0.72(0.63-0.82) | 1.74(1.57-1.91)  0.51(0.42-0.6) |
| Control III | **3.08(2.82-3.34)**  **0.78(0.66-0.89)** | **1.4(1.24-1.56)**  **0.35(0.27-0.43)** | **1.17(1.01-1.32)**  **0.33(0.26-0.39)** |
| Control IV | 3.4(3.17-3.63)  0.84(0.73-0.95) | 1.57(1.42-1.73) 0.39(0.31-0.47) | 1.39(1.25-1.53)  0.37(0.3-0.44) |

TP: Total prevalence, Y_1_: low shedders and Y_2_: high shedders

**References**

1. Pradhan, A. K. et al. Dynamics of endemic infectious diseases of animal and human importance on three dairy herds in the northeastern United States. J. Dairy Sci. 92, 1811–1825 (2009).
2. USDA. Part I: Reference of Dairy Cattle Health and Management Practices in the United States (2007).
3. Lu, Z. et al. The importance of culling in Johne’s disease control. J. Theor. Biol. 254, 135–146 (2008).
4. Al-Mamun, M. A., Smith, R. L., Schukken, Y. H. & Gröhn, Y. T. Modeling of Mycobacterium avium subsp. paratuberculosis dynamics in a dairy herd: An individual based approach. J. Theor. Biol. 408, 105–17 (2016).
5. NAHMS. Dairy 2007–Johne’s Disease on U.S. Dairies, 1991–2007. National Animal Health Monitoring System, Fort Collins, CO (2007).
6. van Schaik, G., Rossiter, C. R., Stehman, S. M., Shin, S. J. & Schukken, Y. H. Longitudinal study to investigate variation in results of repeated ELISA and culture of fecal samples for Mycobacterium avium subsp paratuberculosis in commercial dairy herds. Am. J. Vet. Res. 64, 479–84 (2003).
7. Sweeney, R. W., Whitlock, R. H. & Rosenberger, A. E. Mycobacterium paratuberculosis isolated from fetuses of infected cows not manifesting signs of the disease. Am. J. Vet. Res. 53, 477–80 (1992).
8. 1. IAP: 2005: Estimation of parameters on the vertical transmission of Map in a low-prevalence dairy herd. Available at: http://www.paratuberculosis.info/proc8/abst6_p217.htm. (Accessed: 31st January 2017).
9. 2. IAP: 2005: MAP Super-Shedders: Another factor in the control of Johne’s disease. Available at: http://www.paratuberculosis.info/proc8/abst2_p44.htm. (Accessed: 31st January 2017).
10. Hertl, J. A., Schukken, Y. H., Welcome, F. L., Tauer, L. W. & Gröhn, Y. T. Effects of pathogen-specific clinical mastitis on probability of conception in Holstein dairy cows. J. Dairy Sci. 97, 6942–54 (2014).
11. Cha, E., J. A. Hertl, Y. H. Schukken, L. W. Tauer, F. L. Welcome, and Y. T. Gröhn. The effect of repeated episodes of bacteria-specific clinical mastitis on mortality and culling in Holstein dairy cows J. Dairy Sci. 96, 4993-5007 (2013).\
12. Al-Mamun, M. A. & Grohn, Y. T. MABSDairy: A Multiscale Agent Based Simulation of a Dairy Herd. in Proceedings of the 50th Annual Simulation Symposium 8:1--8:12 (Society for Computer Simulation International, 2017).
13. SAS Institute. 2008. User’s Guide. Version 9.2. SAS Institute Inc., Cary, NC
